# Narrow Leafed Lupin (*Lupinus angustifolius* L.) β-Conglutin Seed Proteins as a New Natural Cytotoxic Agents against Breast Cancer Cells

**DOI:** 10.3390/nu15030523

**Published:** 2023-01-19

**Authors:** Julia Escudero-Feliu, María García-Costela, Sara Moreno-SanJuan, Jose D. Puentes-Pardo, Sandra Ríos Arrabal, Paula González-Novoa, María Isabel Núñez, Ángel Carazo, Jose C. Jimenez-Lopez, Josefa León

**Affiliations:** 1 Biosanitary Research Institute of Granada (ibs.GRANADA), E-18012 Granada, Spain; 2 Cytometry and Microscopy Research Service, Biosanitary Research Institute of Granada (ibs.GRANADA), E-18012 Granada, Spain; 3 Department of Pharmacology, Faculty of Pharmacy, University of Granada, E-18011 Granada, Spain; 4 Department of Radiology and Physical Medicine, Faculty of Medicine, University of Granada, E-18016 Granada, Spain; 5 Biopathology and Regenerative Medicine Institute (IBIMER), Center for Biomedical Research (CIBM), University of Granada, E-18100 Granada, Spain; 6 Department of Stress, Development and Plant Signalling, Estacion Experimental del Zaidin, Spanish National Research Council (CSIC), E-18008 Granada, Spain; 7 UWA Institute of Agriculture, The University of Western Australia, Perth, WA 6009, Australia; 8 UWA School of Agriculture and Environment, The University of Western Australia, Perth, WA 6009, Australia; 9 Clinical Management Unit of Digestive Disease and UNAI, San Cecilio University Hospital, E-18006 Granada, Spain

**Keywords:** breast cancer, nutraceutics, cancer stem cells, natural compounds, chemotherapy, apoptosis, viability, oxidative stress, metastasis, resistance

## Abstract

Breast cancer (BC) is the most widespread tumor in women and the second type of most common cancer worldwide. Despite all the technical and medical advances in existing therapies, between 30 and 50% of patients with BC will develop metastasis, which contributes to the failure of existing treatments. This situation urges the need to find more effective prevention and treatment strategies like the use of plant-based nutraceutical compounds. In this context, we purified three Narrow Leafed Lupin (NLL) β-conglutins isoforms using affinity-chromatography and evaluated their effectiveness in terms of viability, proliferation, apoptosis, stemness properties, and mechanism of action on both BC cell lines and a healthy one. NLL β-conglutins proteins have very promising effects at the molecular level on BC cells at very low concentrations, emerging as a potential natural cytotoxic agent and preserving the viability of healthy cells. These proteins could act through a dual mechanism involving tumorigenic and stemness-related genes such as SIRT1 and FoxO1, depending on the state of p53. More studies must be carried out to completely understand the underlying mechanisms of action of these nutraceutical compounds in BC in vitro and in vivo, and their potential use for the inhibition of other cancer cell types.

## 1. Introduction

Breast cancer (BC) is the most widespread tumor in women and the second type of most common cancer worldwide [1]. BC also represents the second leading cause of cancer-related mortality in developed countries, and the fifth cause of general death worldwide [2]. BC is characterized by an intra and inter-tumoral heterogeneity which is extremely relevant regarding the prognosis, treatment, and perspectives of success of this disease [3]. Tumor location, type of BC, differentiation grade, size, patient age, response or resistance to treatment, and presence of different proteins, such as p53, are also relevant to ensure a better prognosis and personalized treatment for the patient [4].

One of the most common classifications of BC is based on the presence or absence of progesterone receptors (PR), oestrogen receptors (ER), and human epithelial growth factor receptor-2 (HER-2) [5]. This classification divides tumors into luminal A (ER positive) and luminal B (PR positive), basal or triple negative (abbreviated as TNBC, with triple-negative receptors), and HER-2 enriched only subtypes [5]. TNBC is known to be extremely metastatic and resistant to different treatments such as chemo and radiotherapy: in fact, the rates of death and metastasis issues are higher in women with this type of BC [6]. Despite all the technical and medical advances in the existing treatments and prevention strategies [7,8], between 30 and 50% of patients with BC will develop metastasis [9], which contributes to the failure of existing treatments and the poor cure rates worldwide. This can be explained by the intrinsic chemo and radioresistance of some tumors, which is partially caused by the existence of a subpopulation of malignant cancer cells, known as cancer stem cells (CSCs), and their main role in tumor regrowth and spread after the initial treatment [10]. This situation urges the need to find more effective prevention and treatment strategies, focusing not only on better clinical results and survival, but also on preventing side effects and cancer recurrence [11].

According to recent evidence, natural products with nutraceutical properties may be a potentially promising therapeutic strategy for diseases prevention and treatment. In this regard, more resources and research is required to obtain more scientific knowledge and fast advance in this field [12,13,14]. Nowadays, considerable interest is focused on legume seed proteins, particularly those from lupins, a legume of the Fabaceae family [15]. Particularly, the seeds of Narrow-leafed lupin (NLL) *Lupinus angostifolius* L. or blue lupin are attracting attention because of their potential for disease prevention and improvement [16]. These properties are mainly due to their high protein and dietary fiber content [17,18], specifically, the β-conglutin proteins that are the most highly expressed conglutin family in NLL [19]. Seven genes coding for individual β-conglutin proteins, named conglutin β1 to β7, have been described [20].

Recently, the anti-diabetic, antioxidant, and anti-inflammatory action of these β-conglutin proteins was described. These characteristics arise since β1, β3, and β6 conglutins act at different levels, exerting pleiotropic effects on the cells. They are capable of reducing the expression of mRNA of pro-inflammatory mediators, as well as reducing the chemotactic capacity of cells by decreasing the chemokines levels and cell adhesion factors [21,22]. Due to their multiple effects at the molecular level leading to the improvement of inflammatory-based diseases, β-conglutin proteins could be used in the prevention and treatment of inflammatory-related diseases such as obesity, diabetes [21,22] or cancer [23].

Regarding molecular mechanisms of BC progression, SIRT1, as a crucial regulator of cellular targets, is the most studied sirtuin with a promising therapeutic potential for many diseases like cancer, concretely BC [24]. The physiological functions of SIRT1, and particularly in relation with processes like apoptosis or resistance to cancer treatments, are mediated by deacetylation of histones, transcription factors, or co-activators such as p53 or forkhead box O (FOXO) [24]. The SIRT1/FoxO1 regulatory axis is an important pathway implicated in BC progression and aggression [24,25,26,27]. One of the processes regulated by this SIRT1/FoxO1 pathway is autophagy, which has emerged as a crucial and controversial mechanism that can play a dynamic tumor-suppressive or promoting role depending on the cancer context and cellular type [28,29,30]. Recent studies showed that activation of autophagy mechanisms could suppress BC metastasis [29]. The expression of LC3B (microtubule-associated protein 1 light chain 3, MAP1LC3) [31] is of high importance in the autophagy process, and sequesteome 1 (SQSTM1/p62) protein, a classic receptor of autophagy, is usually used as marker for autophagy. p62 degrades itself during the autophagy process [32] and LC3B is incorporated into the membranes of autophagosomes [33]. The interaction between p62 reduced levels and LC3B higher detection can be used to assess autophagy [32].

In this work, we over-expressed and purified β-conglutin proteins and assay them as potential treatments for three BC cell lines a non-tumorigenic one in a 2D breast cell model in vitro. We evaluated the antitumoral properties of these β-conglutins, their potential mechanism of action, and their relationship with the regulation of CSCs phenotype through a different axis that could depend on important BC-related pathways.

## 2. Materials and Methods

### 2.1. Overexpression and Purification of β-Conglutins

The overexpression and purification of these proteins were accomplished following a previously published protocol [21]. Briefly, expression plasmids for each of the β-conglutin isoforms (β1, β3, and β6) were constructed using a modified variant of a pET28a vector (Novagen, Paris) with an N-terminal polyhistidine (6xHis) and a pUC57 vector containing a synthetic gene encoding for each conglutin protein. The overexpression of these β-conglutin isoforms was performed by different expression induction methods in bacteria (*Escherichia coli*) and then the β-conglutin isoforms were purified by a combination of biochemical techniques, (sonication, differential centrifugation, and tandem affinity chromatography) following Qiagen protocol recommendations for His-tagged proteins. Finally, after the design, production, and purification of a highly specific anti-conglutin β antibody (Agrisera, Sweden), the identification of the three β-conglutin proteins previously obtained by means of SDS-PAGE and immunoblotting was performed.

### 2.2. Cell Lines and Culture Conditions

Three human breast cancer cell lines, MDA-MB-231 (high levels of p53 mutant with gaining functionality, triple-negative breast cancer), MCF-7 (p53 wild-type, ERα positive, and weak for HER2), and SK-BR-3 (p53 mutant without gaining functionality, ERα negative, and HER2-positive), and a healthy non-tumorigenic epithelial cell line (MCF-10A), were used. They all were obtained from the ATCC.

Tumoral cell lines were grown in Gibco Dulbecco’s Modified Eagle Medium (DMEM) High-Glucose (Gibco, Carlsbad, CA, USA). MCF-10A cells were grown in DMEM/F-12 (Nutrient Mixture) from Gibco too. Both media were supplemented with 10% fetal bovine serum (FBS), 1% antibiotic cocktail (containing penicillin and streptomycin), 0.25 µg/mL amphotericin B, and 2 mM L-Glutamine. DMEM/F-12 was supplemented with 0.5 µg/mL hydrocortisone, 20 ng/mL epidermal growth factor (EGF), and 10 µg/mL insulin too. Cells were grown at 37 °C in a humidified 5% CO_2_ environment. All cell culture reagents were purchased from Gibco (Carlsbad, CA, USA).

The treatment of the cell lines was carried out using different serial concentrations of β-conglutins 1, 3, and 6, diluted with culture media, from 40 ng/µL to 0.1 ng/µL for 1, 24, 48, and/or 72 h, respectively.

### 2.3. MTT Assay

MTT viability assay was accomplished by seeding 4 × 10^3^ cells per well in 96-well plates, following [34]. Cells were allowed to grow overnight and treated with different concentrations of β-conglutins 1, 3, and 6 for 24, 48, and 72 h.

After conglutin treatment, 10 µL of 5 mg/mL MTT were added to each well and the cells were incubated at 37 °C and 5% of CO_2_ for 4 h. Then, 100 µL of lysis buffer (20% SDS in 50% formamide at pH 4.7) were added to each well. Optical density was measured using the Triad Multimode reader (Dynex Technologies, Chantilly, VA, USA) at 570 nm. Non-treated cells were used as a control. The MTT assay was performed at two different times (24 and 72 h) and concentrations (0–10 µg/mL) of each β-conglutin: β-conglutin 1 (β1), β-conglutin (β3), and β-conglutin (β6). The viability percentage was calculated in comparison with the non-treated (NT) control, assuming a 100% viability for the NT cells.

### 2.4. Trypan Blue Assay

Cells were seeded in 24 well plates (3 × 10^4^ cells/well) and then treated with the β-conglutins for 24 h. After the treatment, cells were trypsinized and resuspended in PBS before staining with Trypan Blue Dye 0.4% from BioRad Laboratories (10 µL cell solution + 10 µL Trypan Blue 0.4%). This assay was performed following the manufacturer’s protocol. After 5 min of incubation, the stained and non-stained cells were counted. Viability was calculated for each condition as (total live cells/total cells) × 100 and the mean percent viability for each cell line and condition was determined by comparing live cells in each condition to the live cells in the non-treated control.

### 2.5. Apoptotic and Ferroptotic Cell Identification

Apoptosis was analyzed using the IP-Annexin V kit (BD Biosciences, Franklin Lakes, NJ, USA). Briefly, 2 × 10^5^ cells were seeded in 6-well plates for each cell line, and after the treatment with β-conglutins, they were trypsinized, washed, and incubated with both AnnexinV-FITC and Propidium Iodide following the manufacturer protocol. Samples were immediately analyzed using the BD FACS Aria IIIu Flow Cytometer (Becton Dickinson, BD Bioscience) from the Cytometry and Microscopy Research Service of the Biosanitary Research Institute of Granada (ibs.GRANADA).

For the ferroptosis assay, cells were incubated for 24 h with Ferrostatin-1, an inhibitor of ferroptosis, diluted 1:1000, and the same protocol was then performed. Ferroptosis percentage was calculated with the difference between the percentage of AnnexinV negative/Propidium Iodide positive cells (necrotic cells) in the non-treated with Ferrostatin-1 condition and the treated ones.

For both apoptosis and ferroptosis, AnnexinV-FITC was detected by a Blue Laser (488 nm) FSC, with a 502 LP (Long Pass) and a 530/30 filter. Propidium Iodide-PI was detected by 561 YGL Laser (561 nm), with a 600 LP and 610/20 filter. Apoptosis was calculated by adding both apoptotic cells (AnnexinV positive/Propidium Iodide negative population) and late apoptotic cells (AnnexinV positive/Propidium Iodide positive population).

### 2.6. ROS Measurement

Intracellular reactive oxidative species (ROS) were monitored seeding 3 × 10^3^ cells/well in a 96-well plate and treating them with β-conglutins (for 24 h) as previously described. Then, intracellular ROS level detection protocol was performed using DCFH-DA (2′7′-Dichlorofluorescein diacetate BioReagent, suitable for fluorescence) for ROS detection (Sigma-Aldrich, St. Louis, MO, USA), following the manufacturer protocol. Briefly, culture medium was removed, cells were washed twice with PBS and then incubated with 100 µL of a 10 μM DCFH-DA concentration in serum-free medium for 30 min in the dark. Finally, fluorescence was measured in a Triad Multimode reader using a wavelength of excitation of 485 nm and a wavelength of emission of 525 nm.

### 2.7. DNA Damage Assay

DNA damage was quantified using the γH2Ax detection kit BD Cytofix/Cytoperm (BD Biosciences, Cat. 554714) and PE-CF594 Mouse anti-H2Ax (pS139) antibody (BD Biosciences, Cat. 564719). Briefly, 2 × 10^5^ cells were seeded in 6-well plates and then treated with the β-conglutins (for 24 h) as described above. Cells were then trypsinized, fixed, and permeabilized following the manufacturer protocol, and finally incubated with the anti-H2Ax antibody for 30 min in the dark. Samples were immediately analyzed by flow cytometry using the BD FACS Aria IIIu Flow Cytometer (Becton Dickinson, BD Bioscience) from the Cytometry and Microscopy Research Service of the Biosanitary Research Institute of Granada (ibs.GRANADA).

For the detection of the γH2Ax population, the PE-Texas Red fluorochrome was detected by a 561 Yellow Green Lase (561 nm), with a 600 LP and 610/20 filter.

### 2.8. Quantification and Characterization of CSCs

To detect aldehyde dehydrogenase 1 (ALDH1) activity (directly related to the number of CSCs) in cell culture with and without treatments, the Aldefluor kit (Stem Cell Technologies) was used according to the protocol proposed by the manufacturer [34]. After the treatments, cells were incubated with BODIPY-amino acetaldehyde (BAAA), a fluorescent non-toxic substrate for ALDH, which was converted into BODIPY-aminoacate (BAA) and retained inside the cells. Viable ALDH1+ cells were quantified by flow cytometry on a BD FACS Aria IIIu Flow Cytometer (Becton Dickinson, BD Bioscience) from the Cytometry and Microscopy Research Service of the Biosanitary Research Institute of Granada (ibs.GRANADA). The specific inhibitor of ALDH, diethylaminobenzaldehyde (DEAB), was used to control for background fluorescence. ALDH1 positive cells were quantified with a FITC fluorochrome that was detected by a Blue Laser (488 nm) FSC, with a 502 LP (Long Pass) and a 530/30 filter.

The characterization of CSCs was performed according to cell surface markers using CD44-PE and CD24-FITC antibodies (Biolegend, San Diego, CA, USA) [34]. After 30 min of incubation in darkness and at 4 °C, the samples were analyzed using a BD FACSAria IIIu flow cytometry (Becton Dickinson, BD Biosciences) from the Cytometry and Microscopy Research Service of the Biosanitary Research Institute of Granada (ibs.GRANADA). CD24-FITC antibody was detected by a Blue Laser (488 nm) FSC, with a 502 LP (Long Pass) and a 530/30 filter. CD44-PE was detected by 561 YGL Laser (561 nm), with a 582/15 filter.

### 2.9. Sphere Formation Assay

Cells were seeded with culture medium in 6 well-plate 2 days before starting the sphere formation assay and then treated for 24 h with the β-conglutins 1, 3, and 6 as previously described. Then, cells were trypsinized and, from each condition, a 12-well triplicate with 1 × 10^2^ cells/well was seeded in ultra-low attachment plates (Corning) in sphere culture medium prepared following [34]: DMEM/F12 supplemented with 1% penicillin/streptomycin, B27 10 µg/mL, 1 µg/mL Hydrocortisone, 4 ng/mL Heparin, 10 ng/mL EGF, and 20 ng/mL FGF, and let grow for 5–8 days. All reagents were purchased from Gibco (Carlsbad, CA, USA). Spheres > 50 µm diameter were counted with Leica DM500 binocular microscope from the Cytometry and Microscopy Research Service of the Biosanitary Research Institute of Granada (ibs.GRANADA).

### 2.10. Western Blot

Trypsinized DPBS cells which were double washed with ice-cold (after the 24 h treatment) and incubated with RIPA lysis and extraction buffer supplemented with protease inhibitors (Santa Cruz Biotechnology). Proteins were quantified using the Bradford assay, denatured, and subsequently separated on SDS-polyacrylamide gels. After the electrophoresis, gels were transferred to PVDF membranes using the Bio-Rad Trans Blot Turbo transfer system (Bio-Rad Laboratories, Inc., Hercules, CA, USA). Membranes were then incubated with the appropriate primary antibodies against SIRT-1, FoxO1, Cleaved and Total Caspase 3, and β-Actin (Abcam, Cambridge, UK) overnight and then secondary antibodies from Santa Cruz Biotechnology, Inc., Dallas, TX, USA, for 1 h. Finally, the proteins were detected using Amersham ECL Select Western Blotting Detection reagent (GE Healthcare, Hatfield, UK) on the membrane and acquiring the images with ChemiDoc MP Imaging System (Bio-Rad Laboratories, Inc., USA). Western Blot images were analyzed and quantified using FiJi software. Densitometry was performed for each membrane and the area and mean intensity of each condition were calculated. Finally, the ratio between the β-actin control and the protein of interest was calculated and compared with the non-treated control ratio in each case.

### 2.11. Autophagy Detection

Autophagy was studied by performing Western Blot (Section 2.10) and incubating for 24 h with both anti-SQSTM1/p62 antibody and anti-LC3B antibody from Abcam, Cambridge, UK. Then, membranes were incubated for 1 h with anti-rabbit and anti-mouse secondary antibodies, respectively, from Santa Cruz Biotechnology, Inc., Dallas, TX, USA. Proteins were detected and images were quantified using the 2.10 protocol. For this concrete experiment, the activation of autophagy was detected if a decreased protein level of p62 was accompanied by an increased protein level of LC3B. The opposite situation describes the downregulation of the autophagy process [32].

### 2.12. Statistical Analysis

All experiments were performed at least in triplicates and the results were expressed as mean ± standard deviation unless otherwise indicated. Statistical analyzes were performed using the Shapiro-Wilk test to analyze the normality of the data set and the One or Two-Way Anova analysis, with Dunnett or Tukey correction, depending on the number of groups and data of each experiment, using Graphad Prism 9.3.0 software. Statistical differences between samples were considered significant when *p* values were *p* < 0.05 (*), *p* < 0.01 (**), or *p* < 0.001 (***). Similarity matrices were performed in Microsoft Excel and XLSTAT programs [35] considering a dissimilarity threshold of 0.95.

## 3. Results

### 3.1. β-Conglutins Inhibit Breast Cancer Cell Lines Growth in a 2D In Vitro Model

**Figure 1:**
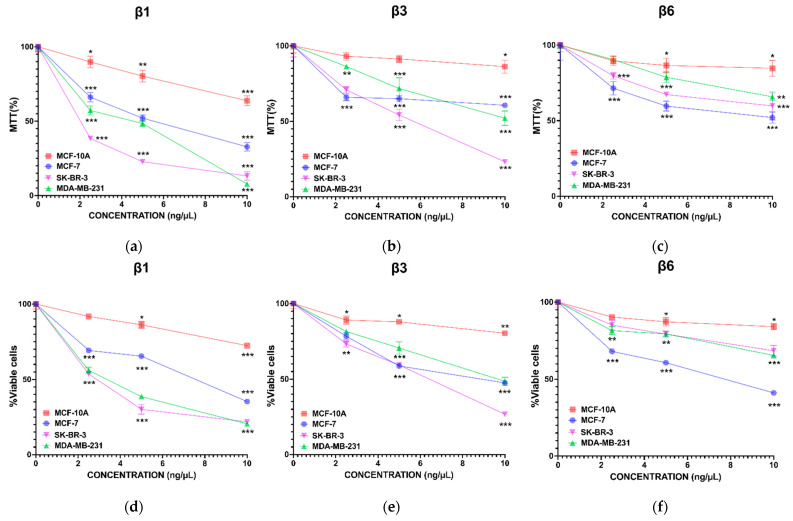
Inhibition of cell growth by β-conglutins in a BC model in vitro. Cells were seeded in 96-well plates at a density of 3 × 10^3^ cells per well. After 72 h of (**a**) β1, (**b**) β3, and (**c**) β6-conglutin treatment, 10 µL of 5mg/mL MTT was added to each well. Absorbance was determined 4 h later for all types of cells. In other experiments, after 72 h of (**d**) β1, (**e**) β3, and (**f**) β6-conglutin treatment, a trypan blue assay was performed to determine the cytotoxicity of treatments versus control (non-treated cells). * *p* < 0.05, ** *p* < 0.01, and *** *p* < 0.001 vs. non-treated cells.

Once the overexpression, quantification, and purification of the β-conglutins were performed, the first objective was to test their potential effect in terms of cell growth in three different BC cell lines (MCF-7, SK-BR-3, and MDA-MB-231) and in a non-tumoral one (MCF-10A). To achieve this, the MTT assay was performed treating cells with serial concentrations of each β-conglutin (0–10 ng/mL) during 24 h (Supplementary Figure S1) and 72 h (Figure 1a–c). After 72 h of treatment, the three β-conglutins had a dose-dependent effect on the BC cell lines treated, with very significant differences in comparison with the healthy cell line MCF-10A, which was more resistant to those treatments. The β1-conglutin induced a very significant effect specially for SK-BR-3 and MDA-MD-231 from very low doses, maintaining the viability of MCF-10A above 85%. Instead, the β3-conglutin was less effective than β1-conglutin in all cell lines, including the non-tumoral one. Finally, β6-conglutin was the least effective in comparison with β1 and β3-conglutins, although it showed a significant effect, especially at higher doses for MCF-7. Therefore, the three β-conglutins studied induced cell growth inhibition more efficiently in the tumor cell lines compared to the non-tumoral one and they also were cytotoxic for BC cells as assayed with the vital dye trypan blue (Figure 1d–f). The effect of these β-conglutins after 24 h of treatment was similar (Supplementary Figure S1). We used these data of viability to construct the proximity matrix. We calculated the Pearson correlation between each pair of β-conglutins. As shown in Table S1, no similarities were found. Similarly, we calculated the Pearson correlation coefficients between each pair of cell line. In this case, we found similarity in the behavior of the MCF-10A and MCF-7 cell lines after treatments (Table S2).

### 3.2. β-Conglutins Induce Caspase-Independent Apoptosis in Breast Cancer Cell Lines

Since β-conglutins clearly affect the viability of BC cell lines, an apoptosis assay was performed for all the cell lines 24 h after treatments with these proteins. Concentrations were chosen based on the cell growth and viability results shown in Figure 1, so that the viability of MCF-10A cells was preserved to the maximum and a significant effect near to a 50% in reducing the viability in at least one of the other three BC cell lines was found.

**Figure 2:**
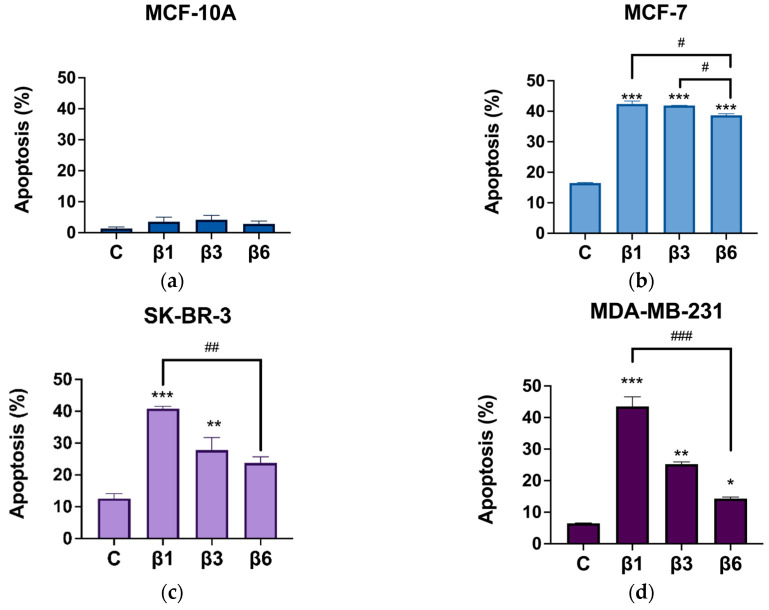
Percentage of apoptotic cells after treatment with β1-conglutin (2.5 ng/µL) β3-conglutin (5 ng/µL) or β6-conglutin (10 ng/µL) for 24 h in (**a**) MCF-10A, (**b**) MCF-7, (**c**) SK-BR-3, and (**d**) MDA-MB-321. After 24 h of (**a**) β1, (**b**) β3, and (**c**) β6-conglutin treatment, cells were trypsinized, washed, and incubated for 15 min with both AnnexinV-FITC and Propidium Iodide from the IP-Annexin V kit (BD Biosciences, UK). Apoptosis was calculated with both early and late apoptotic cells. Samples were immediately analyzed using the BD FACS Aria IIIu Flow Cytometer (Becton Dickinson, BD Bioscience). * *p* < 0.05, ** *p* < 0.01, and *** *p* < 0.001 vs. non-treated cells, and # *p* < 0.05, ## *p* < 0.01, and ### *p* < 0.001 vs. other treatments.

While non-statistically significant changes were found in MCF-10A cells (Figure 2a), β1, β3, and β6-conglutins induced apoptosis in the three BC cell lines, except in SK-BR-3 cells. In this case (Figure 2c), only β1 and β3 induced apoptosis in comparison with the non-treated control. For MCF-7 and MDA-MB-231, the three treatments induce apoptosis, especially in MCF-7 where a 40% of apoptotic cells is reached with β1, β3, and β6 treatments (Figure 2b,d). For the MDA-MB-231cell line, β1 induced the higher percentage of apoptosis in comparison with the other two β-conglutins. Interestingly, neither of the three β-conglutins showed a significant activation of caspase 3 in any of the cell lines tested (Supplementary Figure S2).

**Figure 3:**
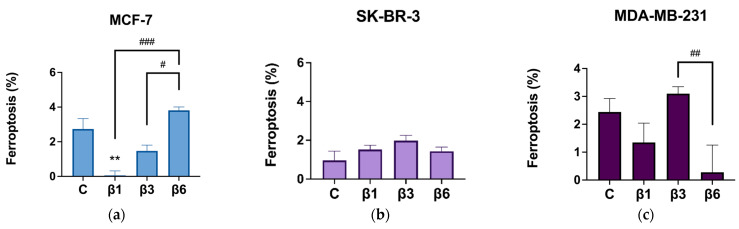
Percentage of ferroptotic cells after treatment with β1-conglutin (2.5 ng/µL), β3-conglutin (5 ng/µL), or β6-conglutin (10 ng/µL) for 24 h in (**a**) MCF-7, (**b**) SK-BR-3, and (**c**) MDA-MB-321. After 24 h of (**a**) β1, (**b**) β3, and (**c**) β6-conglutin treatment +/- Ferrostatin 1 (1 μM) cells were trypsinized, washed, and incubated for 15 min with both AnnexinV-FITC and Propidium Iodide from the IP-Annexin V kit (BD Biosciences, UK). Samples were immediately analyzed using the BD FACS Aria IIIu Flow Cytometer (Becton Dickinson, BD Bioscience). ** *p* < 0.01 vs. non-treated cells, and # *p* < 0.05, ## *p* < 0.01, and ### *p* < 0.001 vs. other treatments.

**Figure 4:**
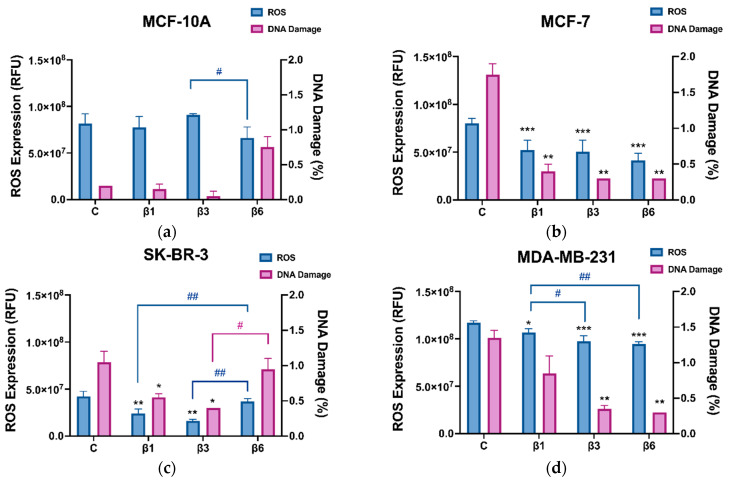
ROS levels and DNA damage percentage after treatment with β1-conglutin (2.5 ng/µL), β3-conglutin (5 ng/µL), or β6-conglutin (10 ng/µL) for 24 h in (**a**) MCF-10A, (**b**) MCF-7, (**c**) SK-BR-3, and (**d**) MDA-MB-321. For intracellular ROS level detection, after 24 h of (**a**) β1, (**b**) β3, and (**c**) β6-conglutin treatment, the culture medium was removed, cells were washed twice with PBS and then incubated with 100 µL of a 10 μM DCFH-DA concentration in serum-free medium for 30 min in the dark. Finally, fluorescence was measured in a Triad multimode reader using a length of excitation of 485 nm and a length of emission of 525 nm. For DNA damage detection, after 24 h of (a) β1, (**b**) β3, and (**c**) β6-conglutin treatment, cells were trypsinized, fixed, and permeabilized following the γH2Ax detection kit BD Cytofix/Cytoperm protocol, and finally incubated with the PE-CF594 Mouse anti-H2Ax (pS139) antibody (BD Biosciences) for 30 min in the dark. Samples were immediately analyzed using the BD FACS Aria IIIu Flow Cytometer (Becton Dickinson, BD Bioscience). * *p* < 0.05, ** *p* < 0.01, and *** *p* < 0.001 vs. non-treated cells, and # *p* < 0.05, ## *p* < 0.01 vs. other treatments.

To elucidate if other caspase-independent death mechanisms activated by β-conglutins could be ferroptosis, the same experiment was performed by adding Ferrostatin-1 (1 μM), an inhibitor of ferroptosis, to each condition during 24 h (Figure 3). MCF-10A cells were not considered for this experiment as they show no activation of apoptosis under those conditions. In general, β-conglutins did not exert any effect on the percentage of ferroptosis, except for β1-conglutin which inhibits this process of cell death in MCF-7 cells (Figure 3a–c). These results are in concordance with the level of ROS and the DNA damage measured after the treatment of cells with these proteins (Figure 4). Representative dot plots for apoptosis and ferroptosis detection by flow cytometry are shown in Figures S3 and S4 of the Supplementary data, respectively.

Treatment of all BC cell lines for 24 h induces an inhibition of ROS levels accompanied by an inhibition of DNA damage. β1 and β3-conlgutins are the most effective treatments for both MCF-7 and SK-BR-3, but β6 has no significant effect on SK-BR-3. Interestingly, no significant changes in ROS levels or DNA damage were found in the healthy cells, MCF-10A. Representative dot plots for DNA damage detection by flow cytometry are shown in Supplementary Figure S5 of the supplementary data.

### 3.3. Implication of SIRT1/FoxO1 Pathway in β-Conglutins Effect on In Vitro Cultured Breast Cancer Cells

**Figure 5:**
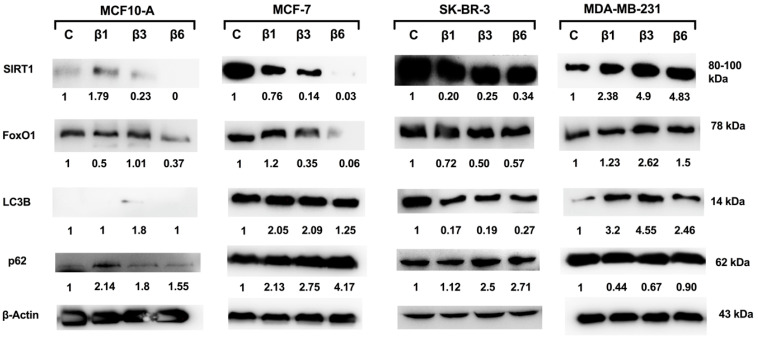
Western blot analysis of SIRT1, FoxO1, LC3B, and p62 proteins. Β-actin was used as a control. Cells were treated with β1-conglutin (2.5 ng/µL), β3-conglutin (5 ng/µL), or β6-conglutin (10 ng/µL) for 24 h in MCF10-A, MCF-7, SK-BR-3, and MDA-MB-321.

In order to elucidate the potential mechanism of action of the β-conglutins, we analyzed changes in SIRT1 expression, implicated in BC growth and progression [26] that is sensible to cellular stress induced by ROS [36,37,38] acting through the activation of FoxO1 transcription factor, among others [39,40]. As shown in Figure 5, treatments with β-conglutins induce a decrease in the expression of SIRT1 in MCF-7 and SK-BR-3 cells. On the contrary, these proteins induce an increase in SIRT1 expression in MDA-MB-231 cells. These changes in SIRT1 expression are accompanied by similar changes in FoxO1 expression.

Since one of the processes regulated by the SIRT1/FoxO1 pathway is autophagy, LC3B and p62 protein expression after treatment was studied (Figure 5). We found an increase of LC3B levels after β1 and β3-conglutin treatment with a decrease of p62 levels in the MDA-MB-231 cell line, which may suggest that autophagy processes are activated under these conditions. As expected, we found decreased expression in LC3B and increased expression of p62 in SK-BR3 cells, indicating inactivation of autophagy. Interestingly, an increase of LC3B levels after β1 and β3 and β6-conglutin treatment with an increase of p62 levels was found in the MCF-7 cell line, which may suggest that autophagy processes are not triggered (up nor down regulation) in this cell line after treatments. Results for MCF-10A showed no significant changes for SIRT1, FoxO1, or LC3B, but a subtle increase in p62, especially after treatment with β1, that is not related to autophagy in this case as it is not accompanied by any significant changes in LC3B expression. Autophagy is not activated for MCF-10A cells.

### 3.4. β-Conglutins Regulate Stemness Phenotype in Breast Cancer

Next, we studied whether β-conglutins regulated stemness in our in vitro model of BC since SIRT1 was identified as a central regulator of progression and metastasis in BC through cancer stem cells (CSCs) and the therapeutic potential of this subpopulation was also described recently [25,34].

**Figure 6:**
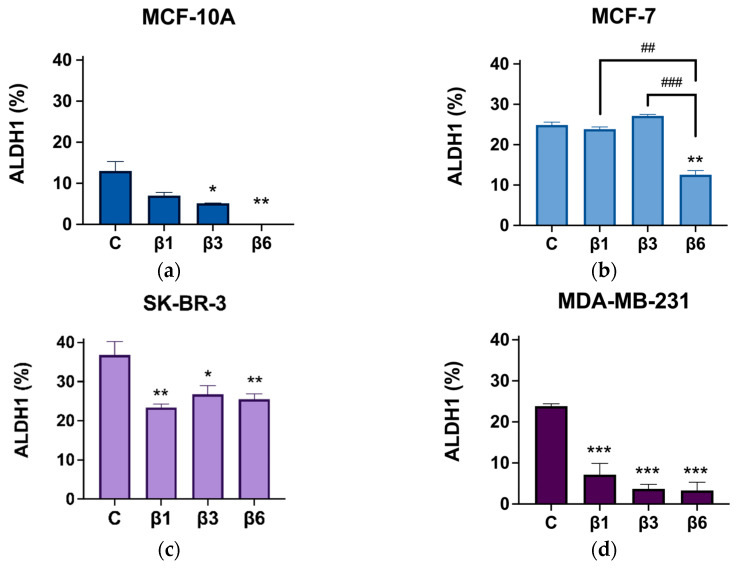
ALDH1 activity after treatment with β1-conglutin (2.5 ng/µL), β3-conglutin (5 ng/µL), or β6-conglutin (10 ng/µL) for 24 h in (**a**) MCF10-A, (**b**) MCF-7, (**c**) SK-BR-3, and (**d**) MDA-MB-321. After 24 h of (**a**) β1, (**b**) β3, and (**c**) β6-conglutin treatment, cells were incubated with BODIPY-amino acetaldehyde (BAAA), a fluorescent non-toxic substrate for ALDH, which was converted into BODIPY-aminoacate (BAA) and retained inside the cells. The specific inhibitor of ALDH, diethylaminobenzaldehyde (DEAB), was used to control for background fluorescence. Viable ALDH1+ cells were quantified by flow cytometry on a BD FACS Aria IIIu Flow Cytometer (Becton Dickinson, BD Bioscience). * *p* < 0.05, ** *p* < 0.01, and *** *p* < 0.001 vs. non-treated cells, and ## *p* < 0.01, and ### *p* < 0.001 vs. other treatments.

Breast CSCs were characterized after treatment with β-conglutins using specific characteristics such as ALDH1 activity and CD44 high/CD24 low expression. The results were compared with a control (non-treated cells). ALDH1 activity (Figure 6) decreased significantly in all cases, except for MCF-7, in which β1 and β3 had no effect (Figure 6b). In addition, β6 was the most effective conglutin in reducing ALDH1 activity in all cell lines tested.

**Figure 7:**
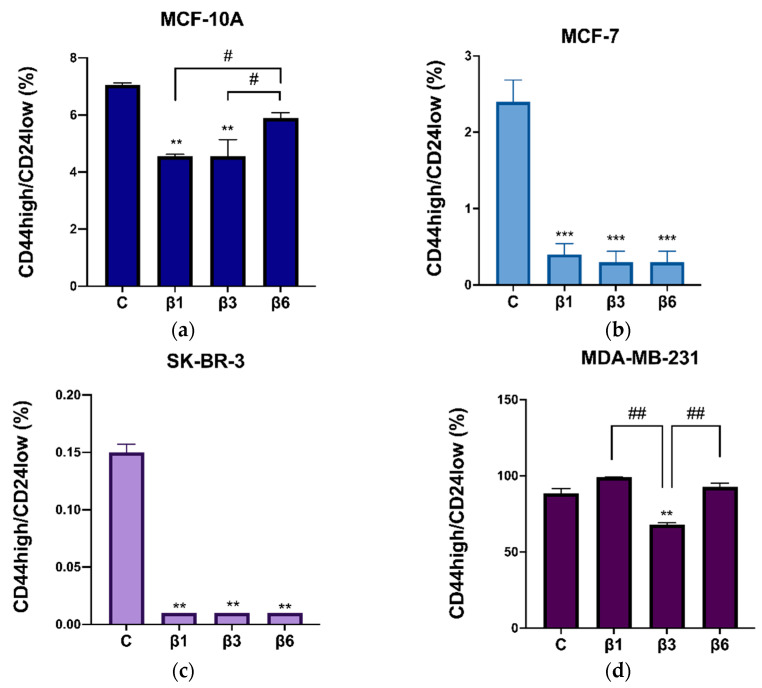
Expression of CD44 high/CD24 low with β1-conglutin (2.5 ng/µL), β3-conglutin (5 ng/µL), or β6-conglutin (10 ng/µL) for 24 h in (**a**) MCF10-A, (**b**) MCF-7, (**c**) SK-BR-3, and (**d**) MDA-MB-321. After 24 h of (**a**) β1, (**b**) β3, and (**c**) β6-conglutin treatment, cells were incubated using CD44-PE and CD24-FITC antibodies (Biolegend, San Diego, CA, USA). After 30 min of incubation in darkness and at 4 °C, the samples were analyzed using a BD FACSAria IIIu flow cytometry (Becton Dickinson, BD Biosciences). ** *p* < 0.01, and *** *p* < 0.001 vs. non-treated cells, and # *p* < 0.05, and ## *p* < 0.01 vs. other treatments.

Similarly to the results obtained for ALDH1 activity, treatments with β-conglutins decreased the expression of the surface markers CD44high/CD24low in the four cell lines tested with comparable efficacy, except for MDA-MB-231 cells. In this case, only β3-conglutin showed a significant decrease in the surface markers (Figure 7). Representative dot plots for both ALDH1 and CD44/CD24 detection by flow cytometry are shown in Figures S6 and S7 of the Supplementary data, respectively.

**Figure 8:**
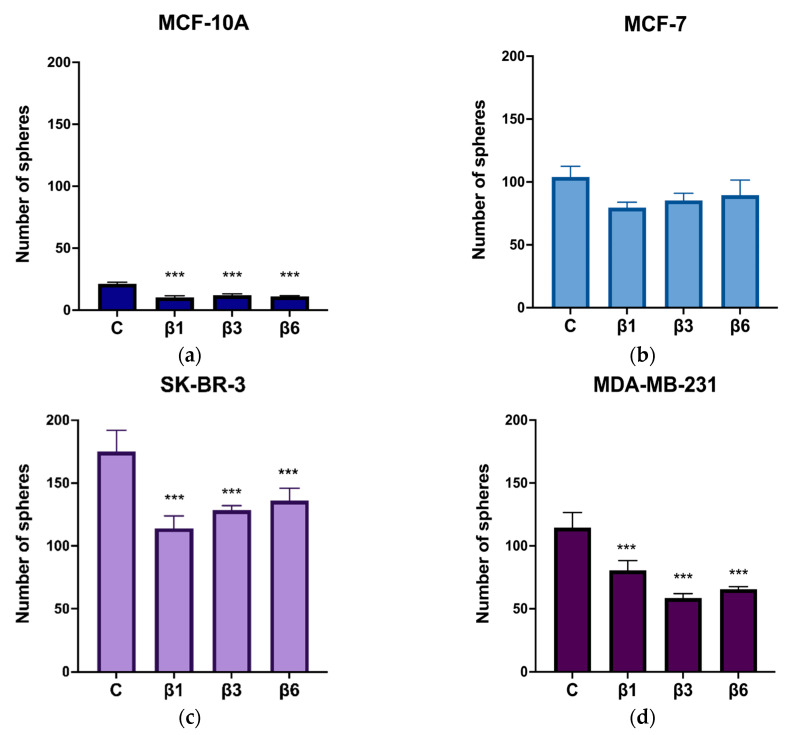

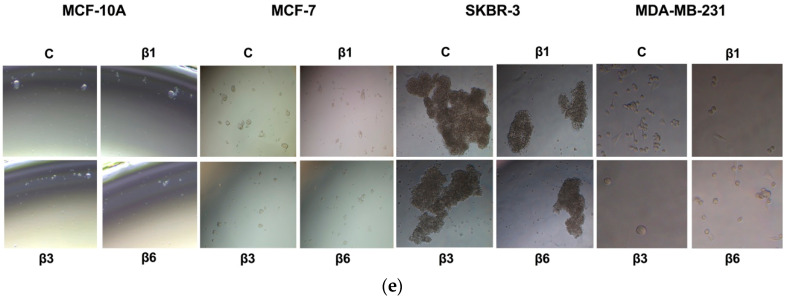
Quantification of mammospheres after treatment with β1-conglutin (2.5 ng/µL), β3-conglutin (5 ng/µL), and β6-conglutin (10 ng/µL). Treatment was performed 24 h before seeding mammospheres from (**a**) MCF-10A, (**b**) MCF-7, (**c**) SK-BR-3, and (**d**) MDA-MB-231 and spheres were characterized in (**e**). After 24 h of (**a**) β1, (**b**) β3, and (**c**) β6-conglutin treatment, cells were trypsinized and, from each condition, a 12 well triplicate with 1 × 10^2^ cells/well were seeded in ultra-low attachment plates in spheres medium (described in 2.9). Spheres > 50 µm were counted and captured using Leica DM500 binocular microscope at 10× magnification. *** *p* < 0.001 vs. non-treated cells.

We also analyzed the self-renewal capacity of cells after β-conglutin treatments through the mammosphere assay. As shown in Figure 8, β1, β3, and β6-conglutins reduced the number of mammospheres in MCF-10A, SKBR-3, and MDA-MB-231 cells. Interestingly, none of the β-conglutins were able to reduce the capacity to form spheres in MCF-7.

## 4. Discussion

To the best of our knowledge, this study is the first attempt to evaluate the molecular effects of NLL β-conglutin proteins in cancer cell lines, concretely in BC ones. Our results showed that β1, β3, and β6-conglutins inhibited cell growth, had a cytotoxic effect, and regulated CSCs reducing stemness properties, especially in cell lines corresponding to the most aggressive BC subtypes (Luminal B and TNBC), preserving the viability of healthy cells, MCF-10A. We proposed a mechanism of action focused on the SIRT1/FoxO1 axis in a p53-dependent manner. In fact, the four cell lines studied the present different status of p53. First, MCF-10A, the epithelial healthy BC cell line is p53 wild-type [41], as well as BC cell line MCF-7 [42]. On the other hand, MDA-MB-231 cells, presenting the most aggressive phenotype type, TN, is a mutant p53-expressing cell line, concretely, the p53-R280K, a gaining function mutation [41,42]. Finally, SK-BR-3 cells also harbored a mutated p53 without gaining functionality, as the mutation is in this case functional p53-E280K [43,44]. Recent evidence has already described different pathways of action and progression of BC cell lines treatments and characteristics depending on p53 status [41,42,45]. Considering the described differences, the recent evidence about the importance of this genetic mutation, and our results for each cell line, it seems that the final effect of the β-conglutins is strongly linked to the state of p53. In fact, a similar effect was found in MCF-7 and MCF-10A cells after β-conglutin treatment, both harboring a wild-type p53, as previously reported [46,47]. Interestingly, the three β-conglutins used did not show similarity between them, indicating that each β-conglutin has a specific behavior and the use of them separately was correct, instead of using the complete β-conglutin protein extract.

NLL conglutins β1, β3, and β6 inhibited the growth and viability of the three BC cell lines studied. This cytotoxic effect is accompanied by increased caspase-independent apoptosis. Other recent research focusing on natural products of isoflavone nature for BC treatment have shown similar effects. Lupiwighteone is present in the root of Glycyrrhiza glabra, a medicinal herb [35], inhibits cell growth for MDA-MB-231 and MCF-7 cells and triggers caspase-independent apoptosis on both of them, whereas epithelial keranocytes were almost unaffected under the same conditions [48]. Another study shows that dioscin, a saponin natural product extracted from *Polygonatum zanlanscianense* suppressed cell growth and induced caspase-independent cell death mechanisms in BC cells [49]. Since many cancer cells have defects in caspase signaling, they can become resistant to conventional chemotherapy drugs that induce caspase-dependent apoptosis [50], so the induction of caspase-independent cell death could be an alternative pathway for overcoming cancer cell resistance.

Among other caspase-independent death cell mechanisms, ferroptosis, an iron-dependent cell death characterized by excess ROS-induced lipid peroxidation [51], has recently emerged as a new cell death mechanism related to the eradication of resistant cancer cells. In fact, recent research has shown a relationship between the inhibition of ferroptosis and the promotion of epithelial-to-mesenchymal transition (EMT), which prompts invasion and is related to the earliest stages of cancer and resistant cells [52]. In our study, β-conglutins reduced ferroptosis in the MCF-7 cell line and induced maintenance of ferroptosis basal levels in SK-BR-3 and MDA-MB-231 cells, indicating that other caspase-independent cell death could be responsible for the effects of these proteins.

Since neither inhibition nor any changes in ferroptosis are found in our study, treatment with β-conglutins could induce similar changes in reactive oxygen species (ROS) levels, as published before [21]. The modulation of ROS in cancer cells may represent a viable strategy in order to overcome drug resistance [53]. Aberrant regulation of redox homeostasis is found in cancer cells compared to normal ones [54]. In fact, other studies have shown that tumor cell lines, especially aggressive ones, such as MDA-MB-231, show higher levels of intracellular ROS compared to luminal or non-tumorigenic breast cells [55], which is in concordance with our results. A recent study with patients showed that the maintenance of chemotherapy-resistant cancer cells in TNBC is due to an increased mitochondrial oxidative phosphorylation and ROS, which is involved in the maintenance of CSCs [56]. Despite all these findings, the role of ROS levels in cancer including BC, remains controversial regarding a therapeutic approach [55]. Both ROS and DNA damage decreased after the β-conglutins treatment for BC cells and maintained their levels for MCF-10A. Increased ROS levels induce DNA mutations that can facilitate cancer metastasis [53], so β-conglutin proteins 1, 3, and 6 could prevent this scenario by inducing a reduction of both ROS and DNA damage, thus affecting viability and inducing cell death in BC cell lines, without affecting those levels in healthy epithelial cells.

The SIRT1/FoxO1 regulatory axis is a ROS-sensitive pathway implicated in BC progression and aggression [24,25,26,27]. This pathway of action was studied using natural compounds such as resveratrol, a dietary phenolic compound which reduced the effectiveness of paclitaxel, one of the usual chemotherapeutics agents in BC, and this reduction was mediated by up-regulation of the SIRT1/FoxO1 pathway in MDA-MB-231 and SK-BR-3 cells [27]. Other studies showed that SIRT1-mediated FoxO1 deacetylation is a key mechanism for multidrug resistance in BC cell lines [48]. Finally, a recent study about the use of an active compound naturally present in many vegetables and medicinal plants, isoalantolactone (IATL) and its anticancer properties in BC showed that this product induced caspase-independent apoptosis that could be related to a ROS-mediated downregulation of SIRT1 [57].

This dual pathway effect also appears in our study: SIRT1 and FoxO1 are up-regulated in MDA-MB-231, and down-regulated in MCF-7 and SK-BR-3. Whereas, no significant changes were found in the healthy epithelial cell line, MCF-10A, supporting our results describing its resistance to this treatment, at least at the doses used in this report. Recent studies investigated the dual effect of the SIRT1/FoxO1 axis as tumor promotor or suppressor in different cancers [58], and p53 has emerged as a downstream effector of this axis in BC [58,59,60]. Previous results showed that in the MCF-7 cell line, with p53 wild-type, the activation of SIRT1 promoted invasion and migration on malignant cells by inhibiting p53 [61]. This matches our results as, in this cell line, the β-conglutins reduced SIRT1 levels which led to reduced cell growth and increased cytotoxicity. On the other hand, other studies have found that high levels of SIRT1 can inhibit tumorigenesis in BRCA-1 BC, which is usually a type of TNBC, and it is the phenotype of the MDA-MB-231 cell line, presenting higher levels of p53 mutations [62,63]. Finally, the cooperation between SIRT1 and p53 could be at the origin of genomic integrity and stability determining its role in cancer progression and aggressiveness [64]. One of the processes regulated by the SIRT1/FoxO1 pathway is autophagy. Recent studies have described this process as a precursor of apoptosis [30], and it can either inhibit or collaborate with apoptosis in tumor therapy. Our results showed that only in TNBC phenotype-like cells, MDA-MB-231, with mutant p53 gaining functionality, SIRT1 and FoxO1 increase is accompanied with autophagy induction. Other natural compounds have already described this pathway of action for their anti-cancer effects, such as Eugenol, a promising anti-cancer agent against TNBC and HER-2 positive BC (MDA-MB-231 and SK-BR-3, respectively), that targets the caspase pathway and induce autophagic cell-death [65]. As expected, inhibition of autophagy was observed in SKBR-3 cells after β-conglutin treatment since it induced downregulation of the SIRT/FoxO1 pathway in these cells. However, in the MCF-7 cells, the treatment induced inhibition of SIRT/FoxO1 but not changes in autophagy. It was previously reported that the genic knock-out of SIRT1 reduced the proliferation, migration, and invasion of MCF-7 breast cancer cells [61]. In addition, a recent report showed that, for this specific cell line, MCF-7, the up-regulation of the SIRT1/FoxO1 pathway causes the induction of ferroptosis in a p53-dependent manner [66]. Those results suggest that, at least after the β1-conglutin treatment, the mechanism of action in MCF-7 could be induced by the regulation of the ferroptotic process instead of the regulation of autophagy observed for the other two BC cell lines. Ultimately, it seems that the effect of the β-conglutin treatment is strongly linked to the status of p53. These proteins could modulate the duality of SIRT1/FoxO1 pathway, leading to an up or down-regulation of autophagy with different mutant p53 statuses or a ferroptosis down-regulation for p53 wt. However, more experiments with different cell lines are necessary to corroborate these results.

**Figure 9:**
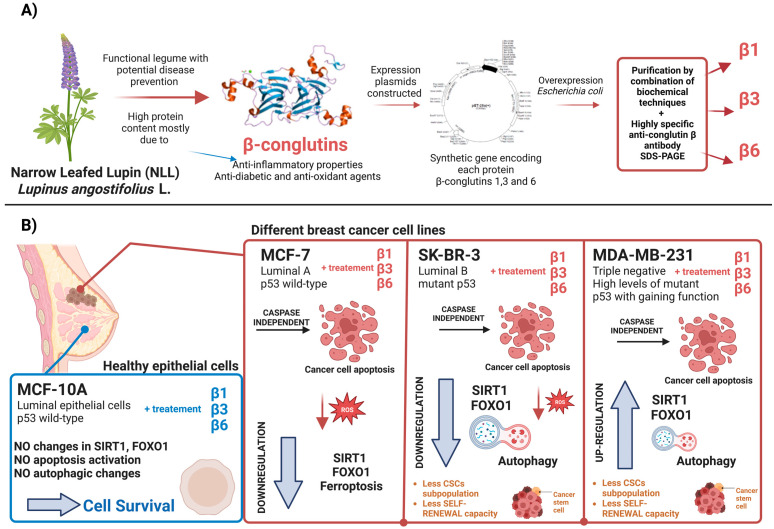
(**A**) Obtention, purification, and application of β-conglutin treatment in BC cell lines as a cytotoxic and anti-stemness agent against BC cell lines, preserving healthy epithelial cells. (**B**) Regulation of SIRT1/FoxO1 pathway and autophagy in a p53-dependent manner. Created with BioRender.com.

The SIRT1 signaling pathway plays a key role in the regulation of genes related to metastasis and stemness in BC [58]. In fact, Shi et al. demonstrated that SIRT1-centered circuitry regulates CSCs origination, related to distant-metastasis and drug-resistance in BC [26]. Other recent investigations suggest that autophagy, regulated by SIRT1/FoxO1 axis, plays a dual role in maintaining the activity of breast CSCs and could emerge as a therapeutic target in association with apoptosis [67]. All this evidence supports the relation between SIRT1 pathway and regulation of CSCs, resistance, and metastasis. Our results showed that β1, β3, and β6 have anti-stemness properties, reducing the number of CSCs and their phenotype in the three BC cell lines. Interestingly, the mammosphere characterization showed that the capacity of self-renewal was only reduced in the TNBC phenotype-like cell line (MDA-MB-231) and the HER-2 positive cell line (SKBR-3), that are representative of BC tumors with worst prognosis and more aggressivity than the luminal A cell line, MCF-7 [5,6,8,11], in which the β-conglutins treatment had no significant effect. This could suggest that β-conglutins are only effective in terms of stemness regulation when autophagy is regulated in BC cell lines, being an effective CSCs targeted treatment for Luminal B and TNBC phenotype-like cell lines. Finally, β-conglutins reduced stem-cell like properties in the healthy epithelial cell line too. This could be a preventive strategy against cell malignant transformation. As described in recent studies, the epithelial-to-mesenchymal transition (EMT) induction in healthy MCF-10A cells contributed to acquisition of stem-like character, increasing CD44+ /CD24− percentage and mammosphere forming capacity [68]. Our treatment seems to generate the opposite effect, that could potentially be related to a prevention from EMT, directly related to malignant invasion and the earliest stages of cancer [68]. Figure 9 summarizes both the process of obtention and purification of the NLL β-conglutins and the results obtained after the treatments of different breast cell lines.

## 5. Conclusions and Perspectives

The main finding of this study is the fact that NLL β-conglutins, which had never been studied in cancer cell lines, can become a cytotoxic agent of great interest, particularly in BC cell lines. They could also be sensitizers for other existing drugs and treatments due to their effect on the stem cells, and even prevention targets for healthy cells, regulating EMT and malignant transformation, and reducing the probability of metastasis and recurrence in tumoral BC cell lines. β-conglutins modulate the effect of SIRT1/FoxO1 pathway, depending on both the status of p53 and the tumor phenotype (luminal, HER-2 positive, or TNBC), a mechanism that could trigger cancer resistance and metastasis. The mechanism of cell death ligated to the status of p53 after β-conglutin treatment seems to be crucial for the final regulation of the phenotype and auto-renovation ability of CSCs in BC in vitro. However, more studies after this initial approach must be carried out to completely understand the therapeutic potential and mechanism of action of these nutraceutical compounds in breast cancer in vitro and in vivo, and their potential use in treatment and prevention for other cancers.

## Data Availability

Not applicable.

## Supplementary Materials

The following supporting information can be downloaded at: https://www.mdpi.com/article/10.3390/nu15030523/s1, Figure S1: Inhibition of cell growth by β-conglutins in a BC model in vitro 24 h post treatments; Figure S2: Western Blot analysis of Cleaved caspase 3 and total Caspase 3; Figure S3: Representative dot plots for apoptosis results; Figure S4: Representative dot plots for ferroptosis results; Figure S5: Representative dot plots for DNA Damage results; Figure S6: Representative dot plots for ALDH1 results; Figure S7: Representative dot plots for CD24/CD44 surface markers results; Table S1: Similarity matrix for paired β-conglutins treatments and Table S2: Similarity matrix for paired cell lines treated.
