# Supplementary material for: Narrow Leafed Lupin (Lupinus angustifolius L.) β-Conglutin Seed Proteins as a New Natural Cytotoxic Agents against Breast Cancer Cells
Source: Nutrients. 2023 Jan 19;15(3):523. doi: 10.3390/nu15030523 (PMC9919070; doi:10.3390/nu15030523)
Supplement: Supplementary file 1 [file nutrients-15-00523-s001.zip › nutrients-2141530-supplementary.pdf]

Supplementary matherial

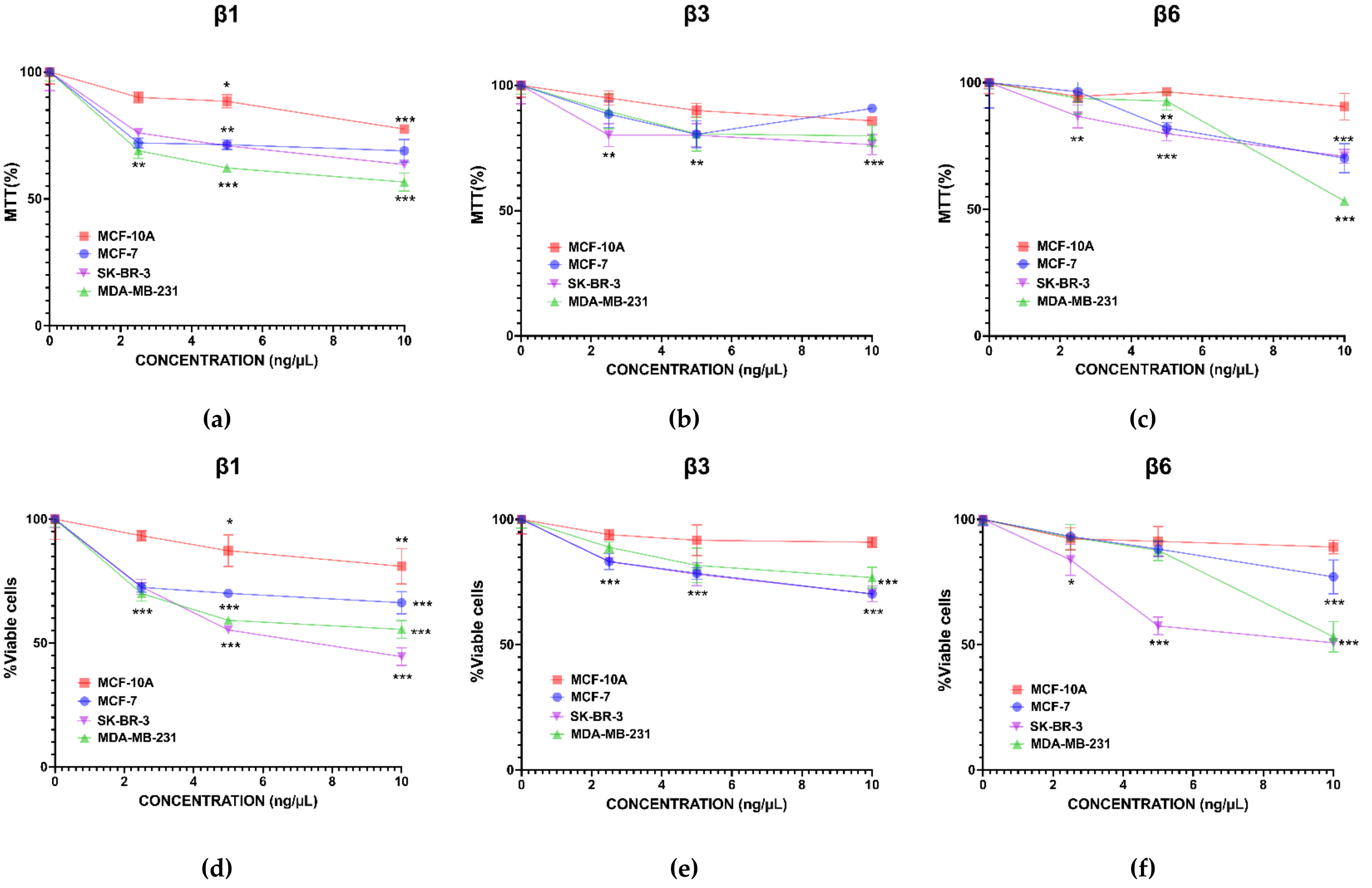

Figure S1. Inhibition of cell growth by β-conglutins in a BC model *in vitro*. Cells were seeded in 96-well plates at a density of 3x10^3 cells per well. After 24 hours of (a) β1, (b) β3, and (c) β6-conglutin treatment, 10 µl of 5mg/ml MTT were added to each well. Absorbance was determined 4 h later for all types of cells. In other experiments, after 24 hours of (d) β1, (e) β3, and (f) β6-conglutin treatment, trypan blue assay was performed to determine the cytotoxicity of treatments versus control (non-treated cells). *$p<0.05$, **$p<0.01$, and ***$p<0.001$ vs non-treated cells.

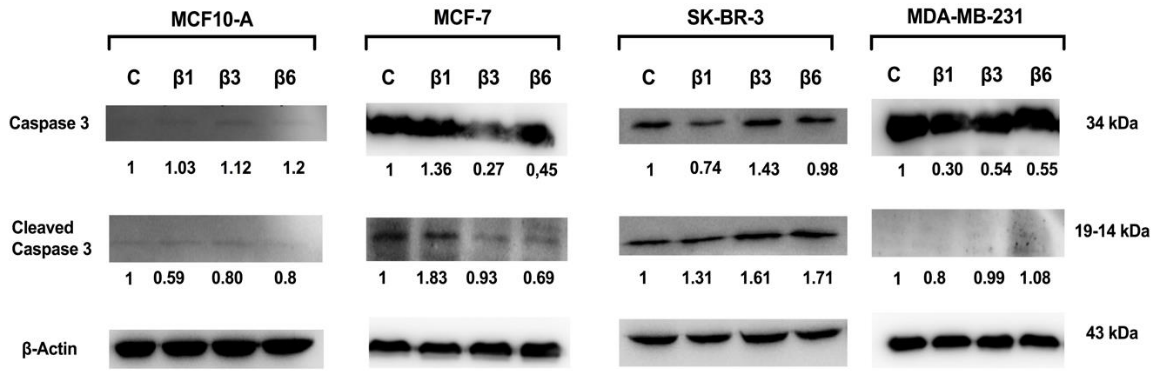

Figure S2. Western Blot analysis of Cleaved caspase 3 and total Caspase 3. β-actin was used as a control**.** Cells were treated with β1-conglutin (2.5 ng/μl), β3-conglutin (5 ng/μl) or β6-conglutin (10 ng/μl) for 24h in MCF10-A, MCF-7, SK-BR-3 and MDA-MB-321.

MCF-10A

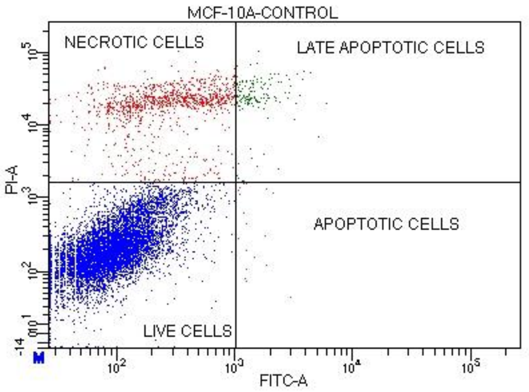

(a)

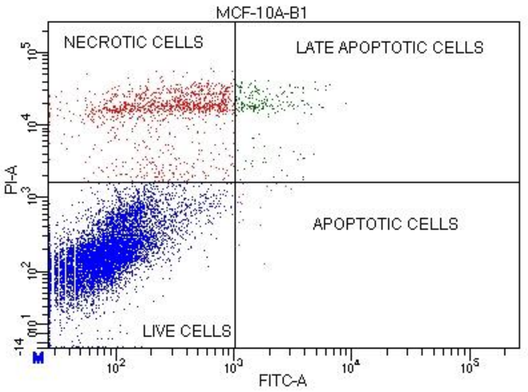

(b)

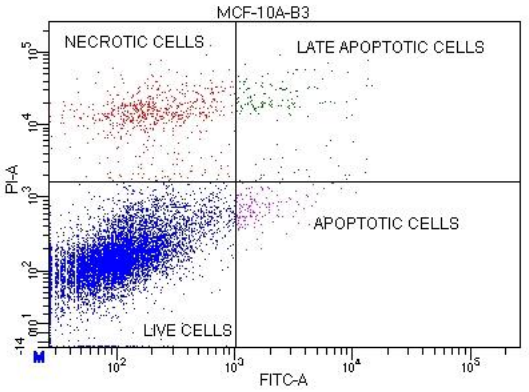

(c)

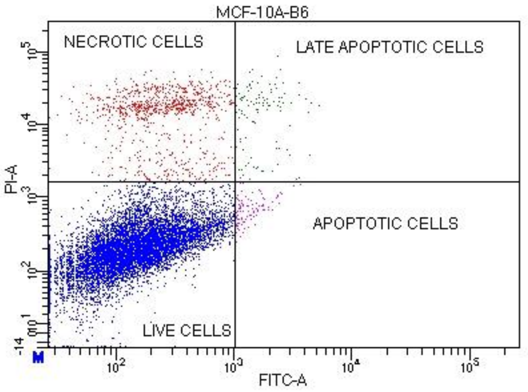

(d)

MCF-7

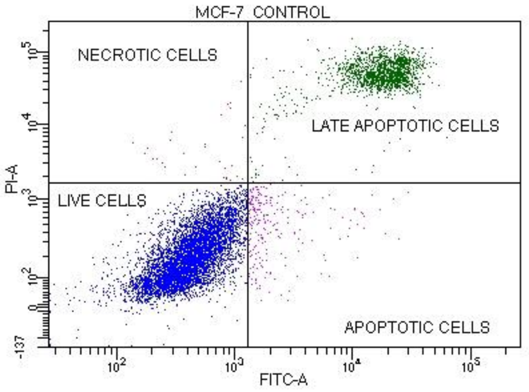

(e)

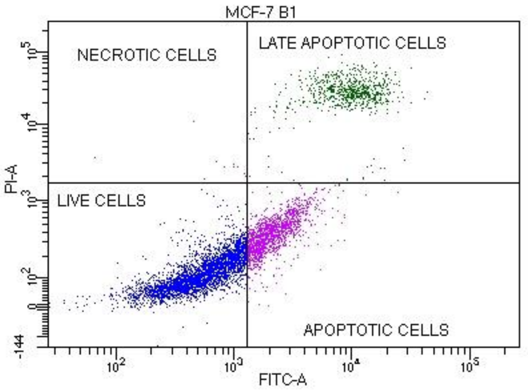

(f)

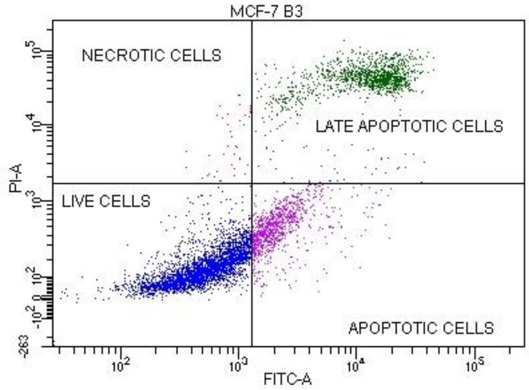

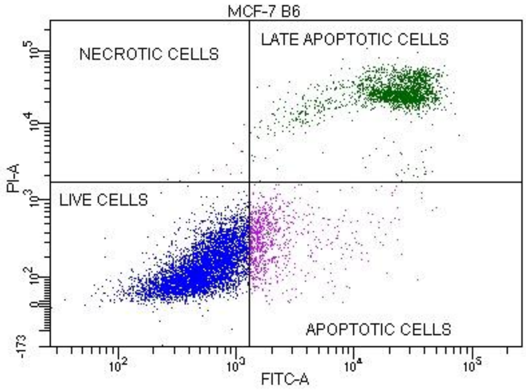

(g) **(h)**

SK-BR-3

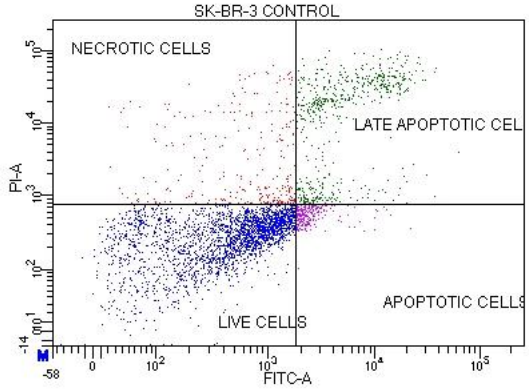

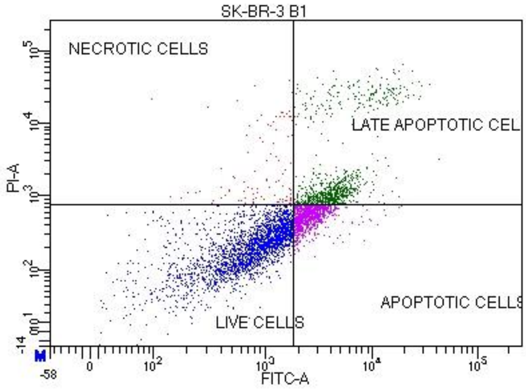

(i) **(j)**

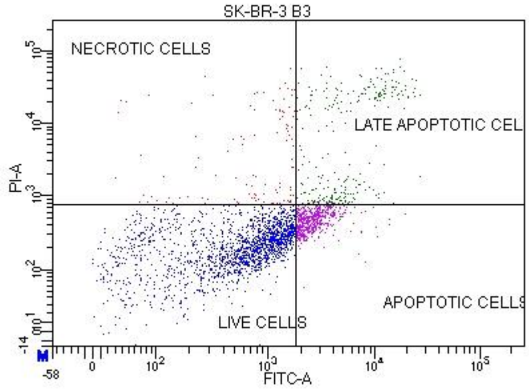

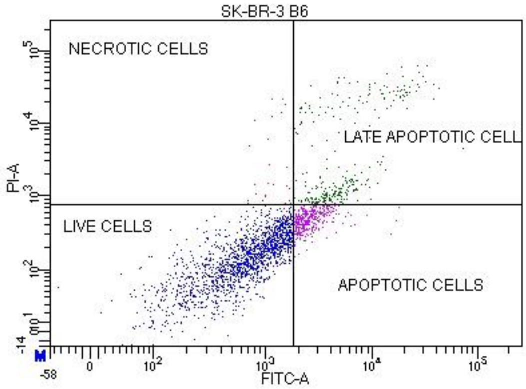

(k) **(l)**

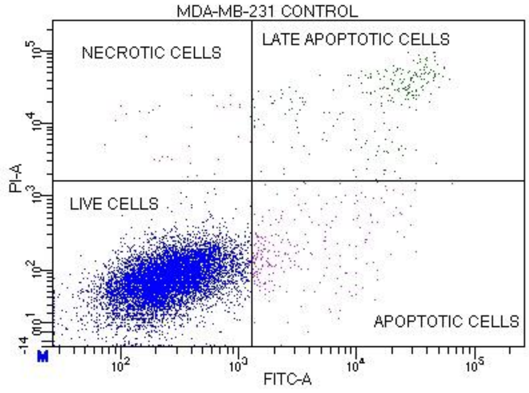

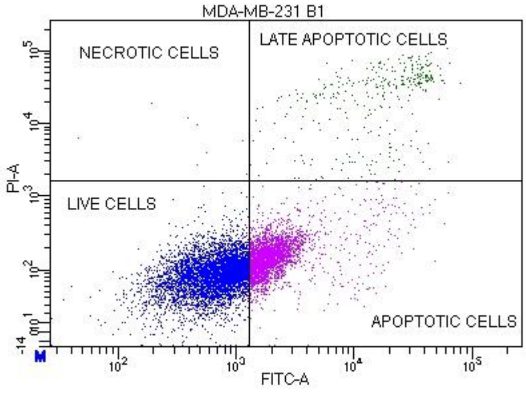

(m) **(n)**

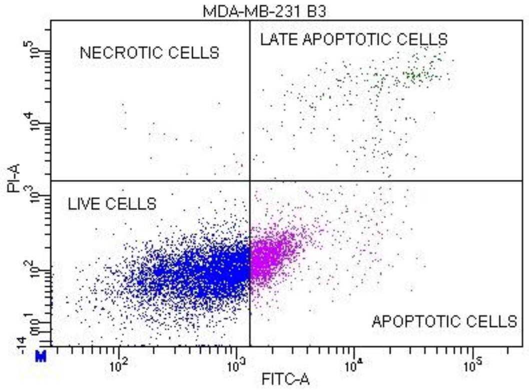

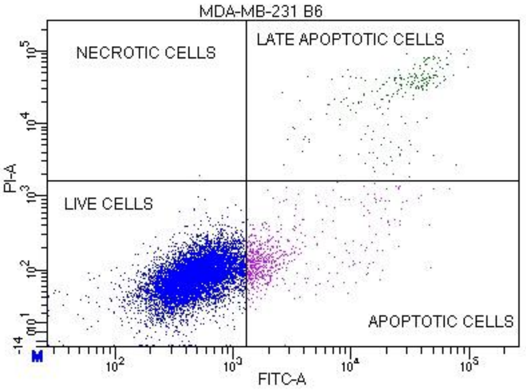

(o) **(p)**

Figure S3. Representative dot plots for apoptosis results. Cells were treated with β1-conglutin (2.5 ng/μl), β3-conglutin (5 ng/μl) or β6-conglutin (10 ng/μl) for 24h in MCF10-A (a-d), MCF-7(e-h), SK-BR-3 (i-l) and MDA-MB-321(m-p). After 24 hours of β1, β3, and β6-conglutin treatment, cells were trypsinized, washed and incubated for 15 min with both AnnexinV-FITC and Propidium Iodide from the IP-Annexin V kit (BD Biosciences, UK). Samples were immediately analyzed using the BD FACS Aria IIIu Flow Cytometer (Becton Dickinson, BD Bioscience).

MCF-7

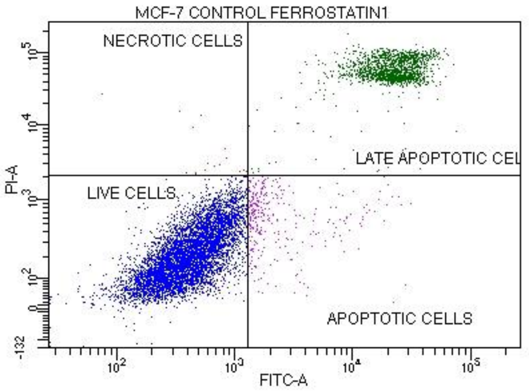

(a)

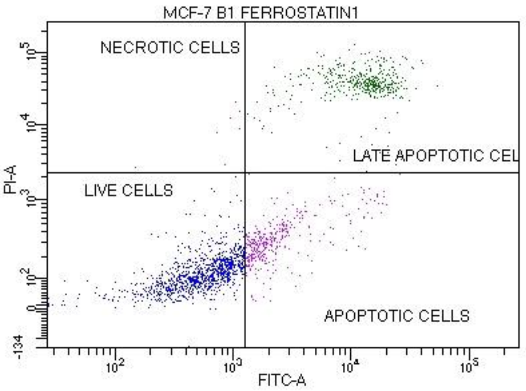

(b)

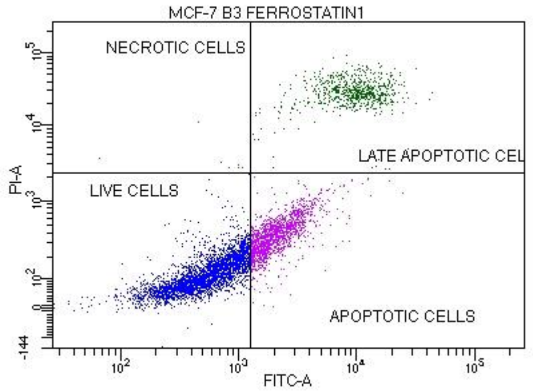

(c)

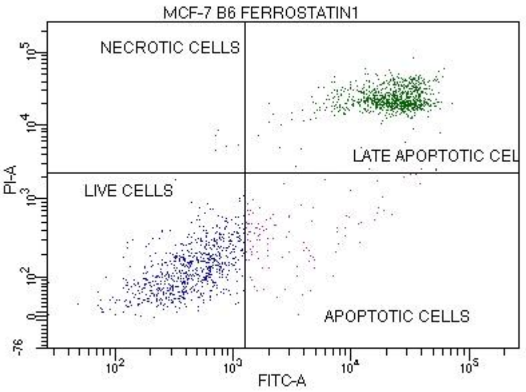

(d)

SK-BR-3

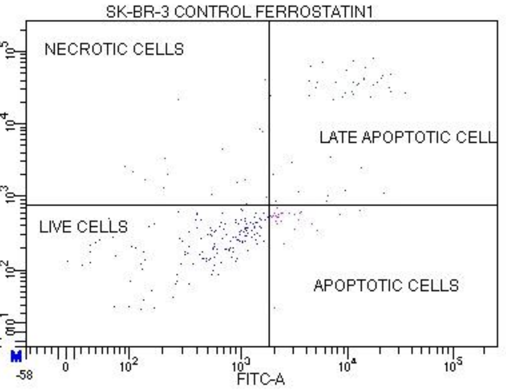

(e)

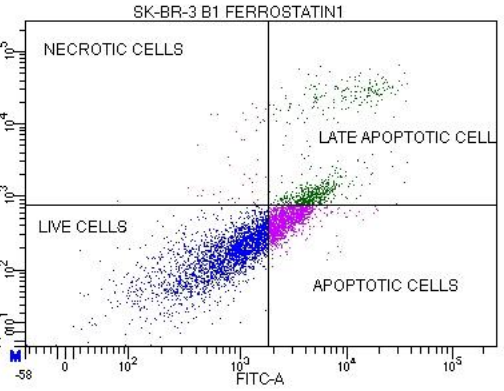

(f)

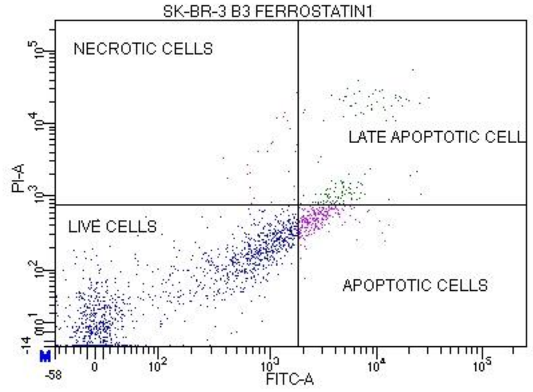

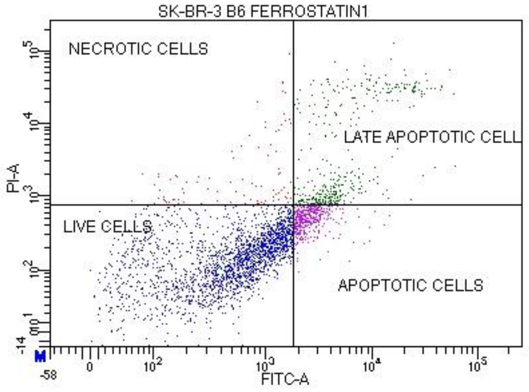

(g) **(h)**

MDA-MB-231

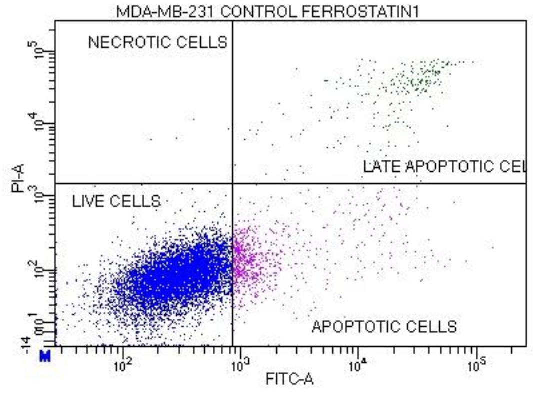

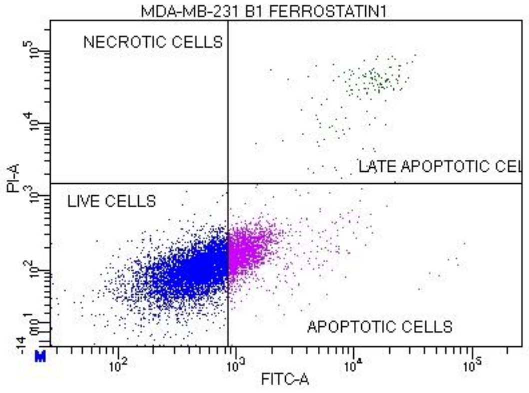

(i) **(j)**

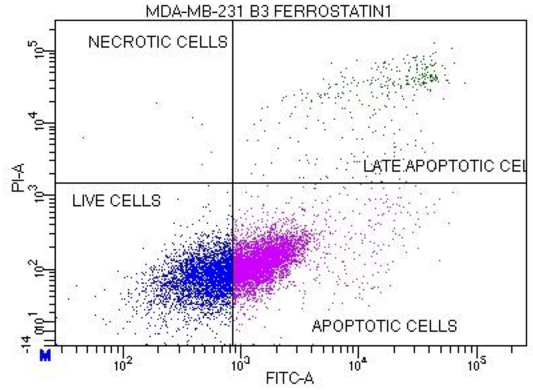

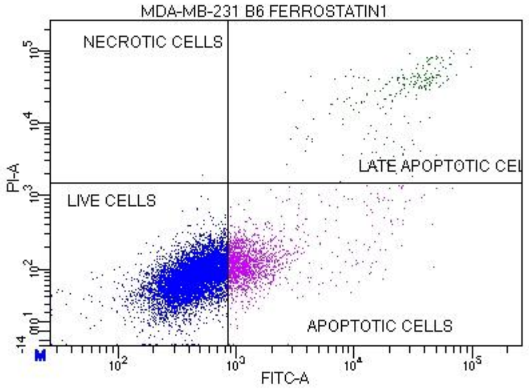

(k) **(l)**

Figure S4. Representative dot plots for ferroptosis results. Cells were treated with β1-conglutin (2.5 ng/µl), β3-conglutin (5 ng/µl) or β6-conglutin (10 ng/µl) for 24h in MCF-7 (a-d), SK-BR-3 (e-h) and MDA-MB-321 (i-l). After 24 hours of β1, β3 and β6-conglutin treatment +/- Ferrostatin 1 (1 µM), cells were trypsinized, washed and incubated for 15 min with both AnnexinV-FITC and Propidium Iodide from the IP-Annexin V kit (BD Biosciences, UK). Samples were immediately analyzed using the BD FACS Aria IIIu Flow Cytometer (Becton Dickinson, BD Bioscience). Ferroptosis percentage was calculated with the difference between the percentage of AnnexinV negative/Propidium Iodide positive cells (necrotic cells) in the

non-treated with Ferrostatin-1 condition (Figure S3, Apoptosis) and the treated ones.

MCF-10A

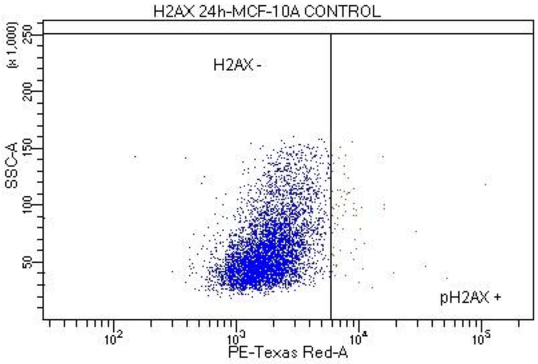

(a)

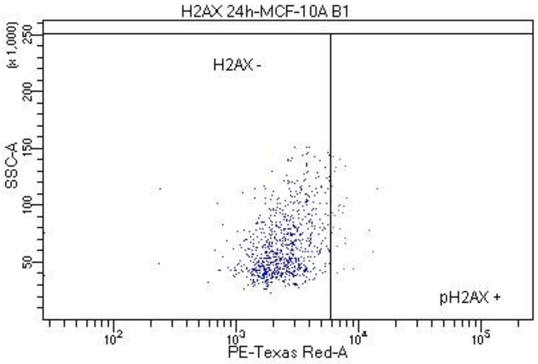

(b)

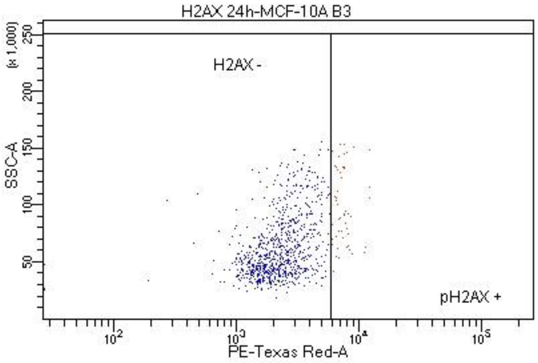

(c)

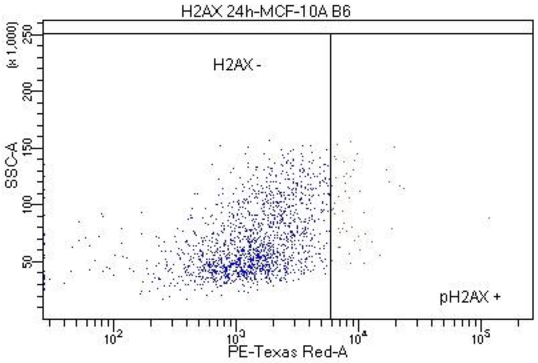

(d)

MCF-7

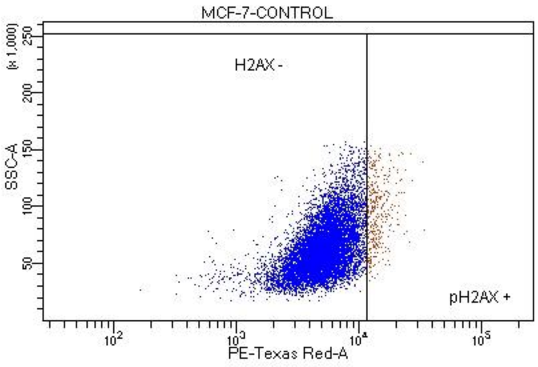

(e)

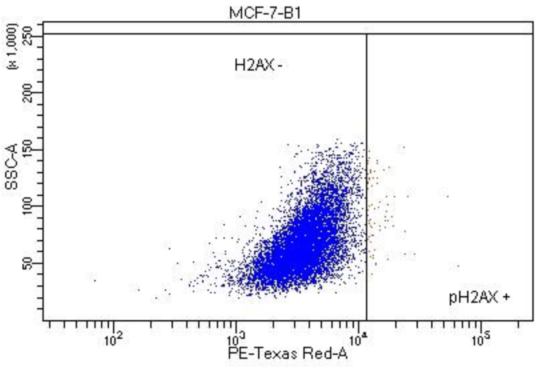

(f)

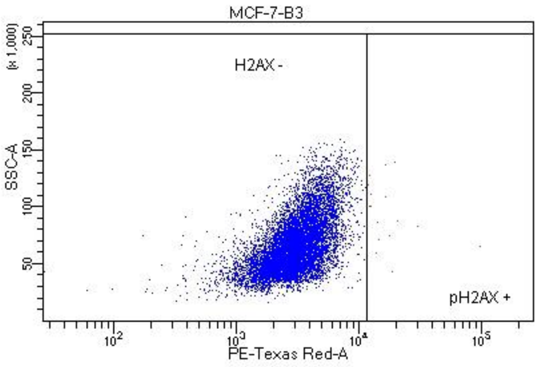

(g)

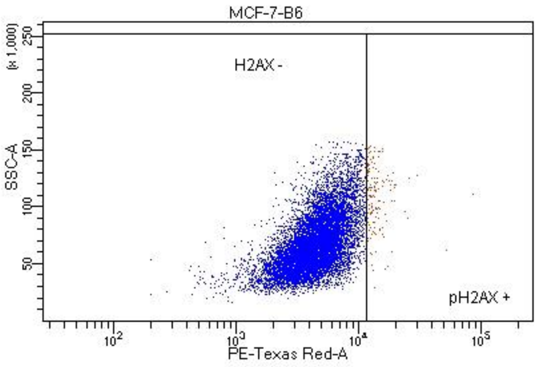

(h)

SK-BR-3

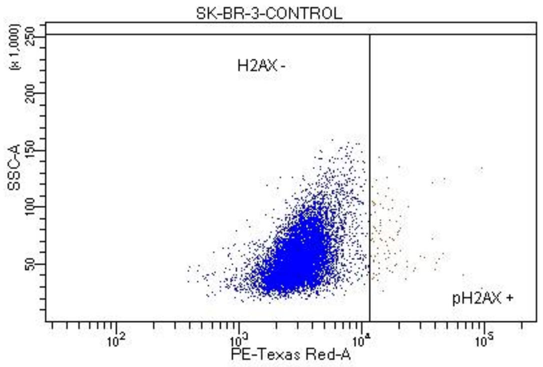

(i)

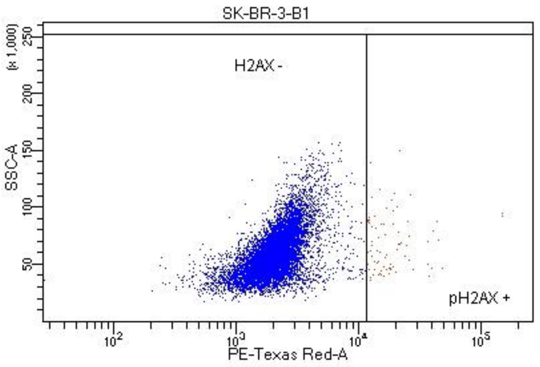

(j)

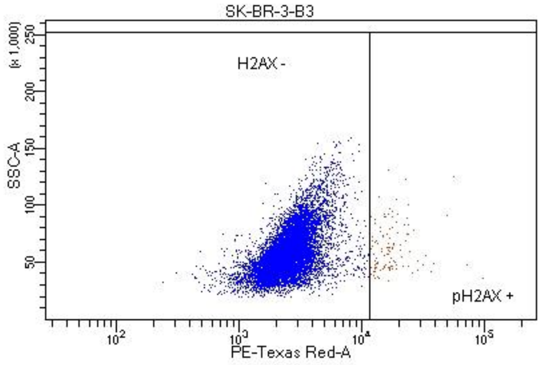

(k)

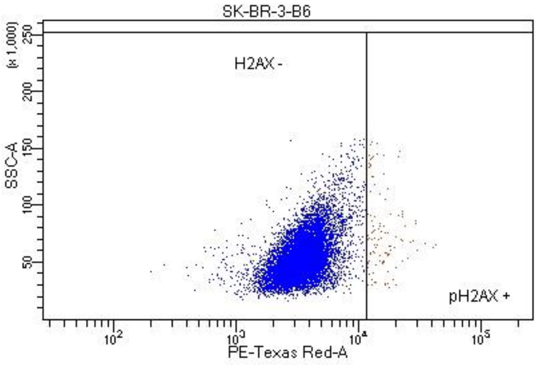

(l)

MDA-MB-231

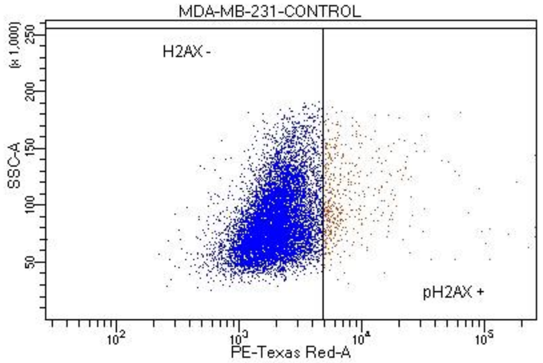

(m)

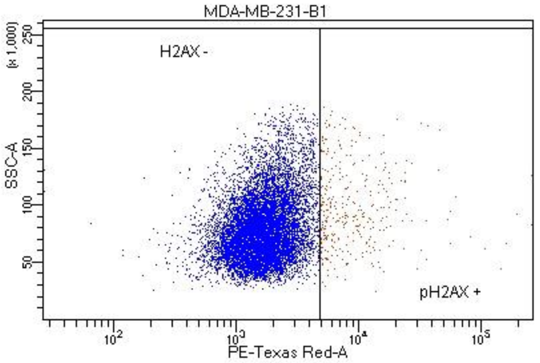

(n)

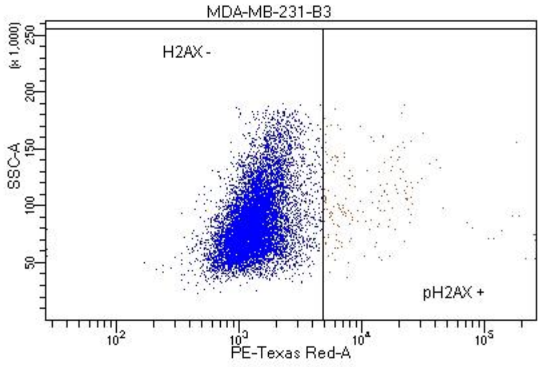

(o)

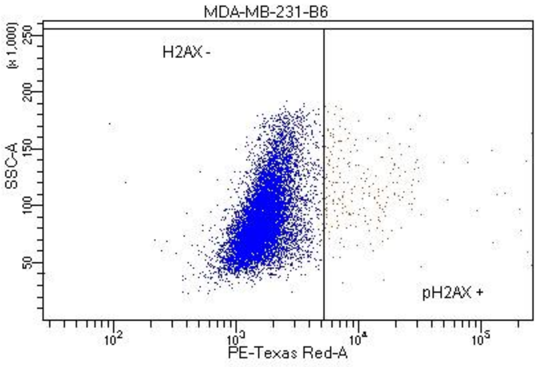

(p)

Figure S5. Representative dot plots for DNA Damage results. Cells were treated with β1-conglutin (2.5 ng/μl), β3-conglutin (5 ng/μl) or β6-conglutin (10 ng/μl) for 24h in MCF10-A (a-d), MCF-7 (e-h), SK-BR-3 (i-l) and MDA-MB-321 (m-p). For DNA Damage detection, after 24 hours of β1, β3 and β6-conglutin treatment, cells were trypsinized, fixed and permeabilized following the γH2Ax detection kit BD Cytofix/Cytoperm protocol, and finally incubated with the PE-CF594 Mouse anti-H2Ax (pS139) antibody (BD Biosciences) for 30 minutes in the dark. Samples were immediately analyzed using the BD FACS Aria IIIu Flow Cytometer (Becton Dickinson, BD Bioscience).

MCF-10A

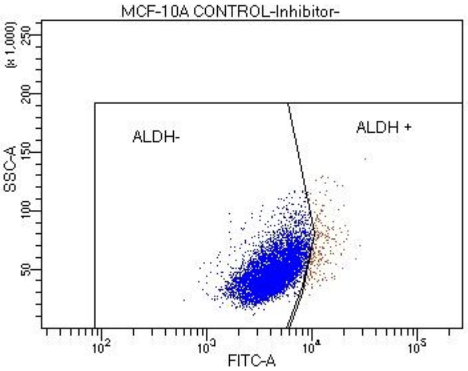

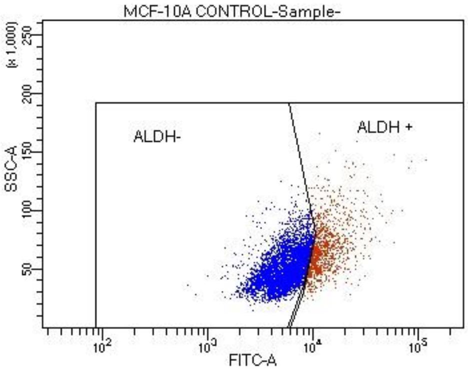

(a)

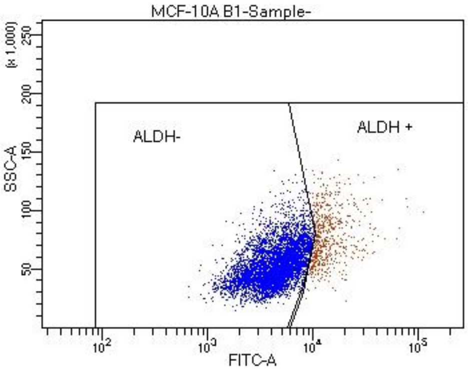

(b)

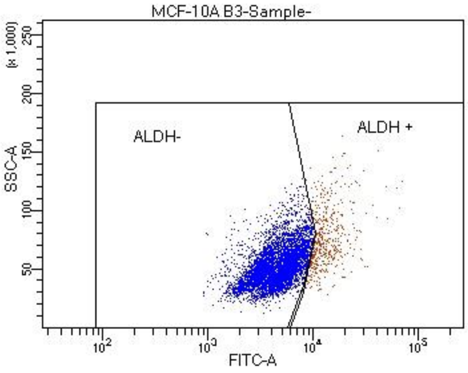

(c)

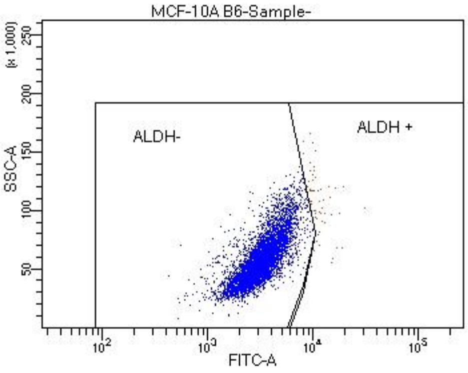

(d)

MCF-7

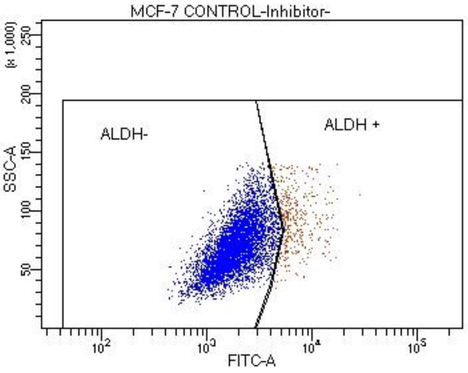

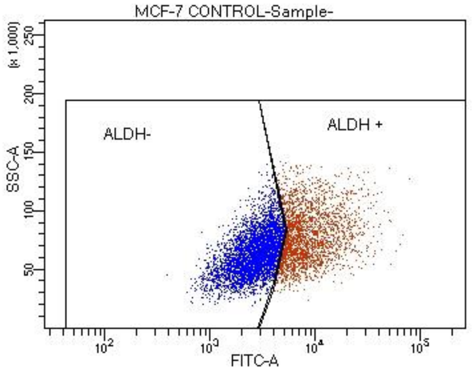

(e)

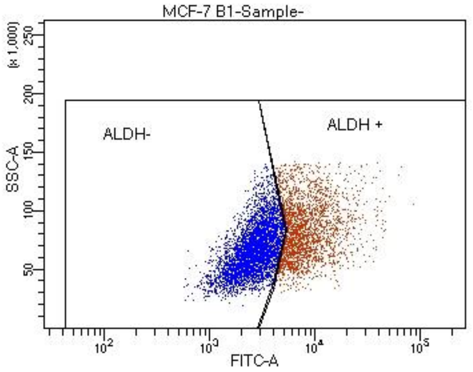

(f)

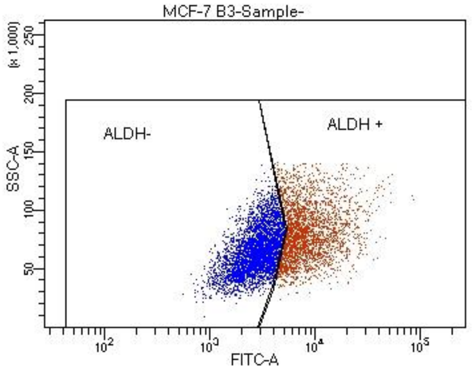

(g)

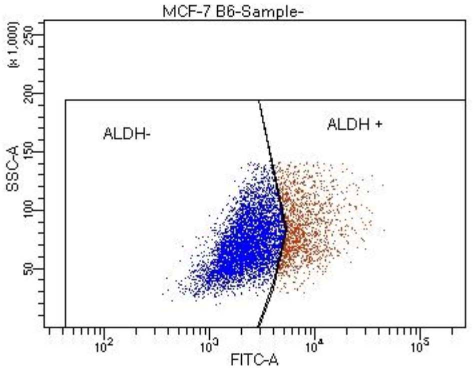

(h)

SK-BR-3

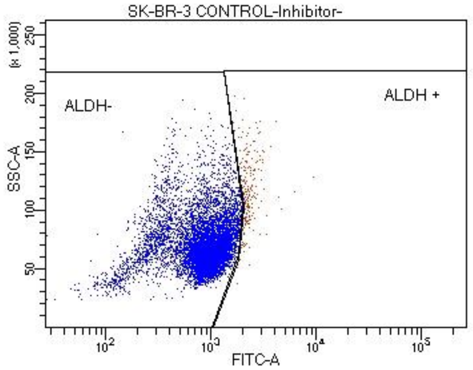

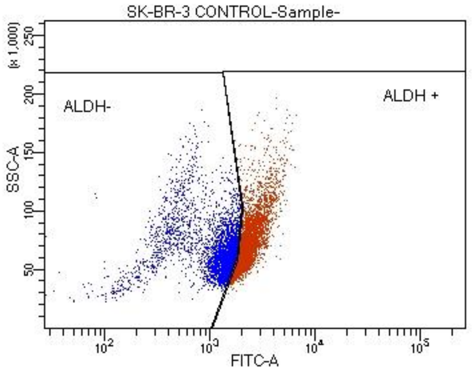

(i)

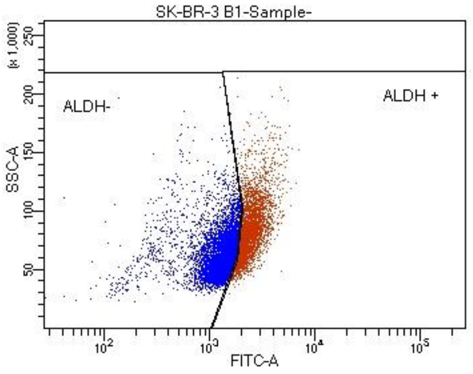

(j)

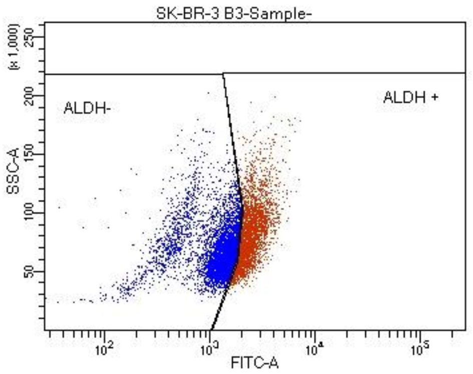

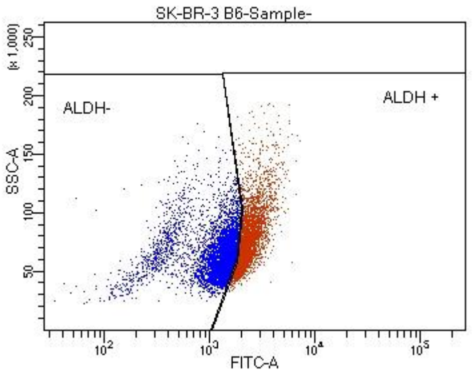

(k) **(l)**

MDA-MB-231

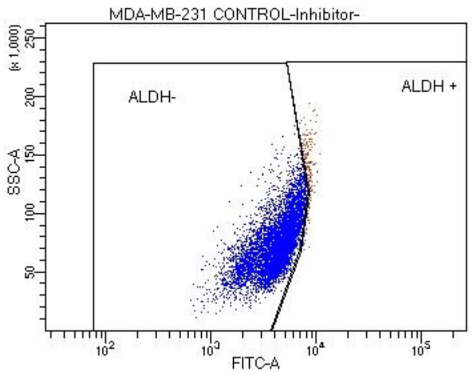

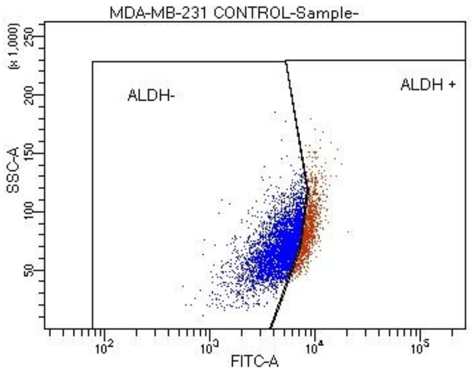

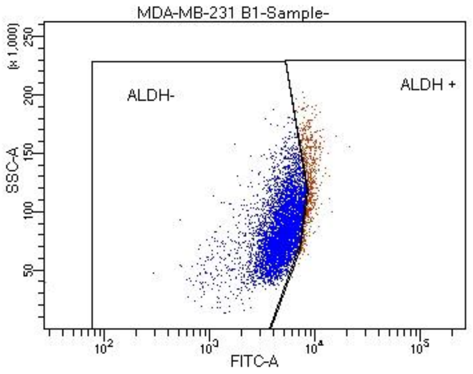

(m) **(n)**

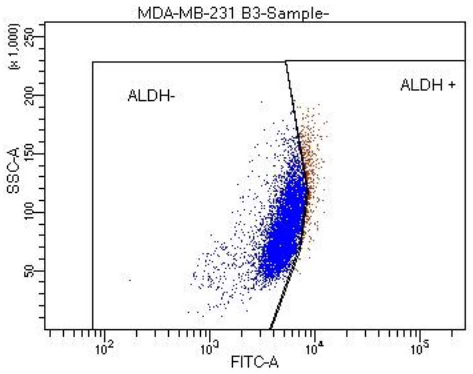

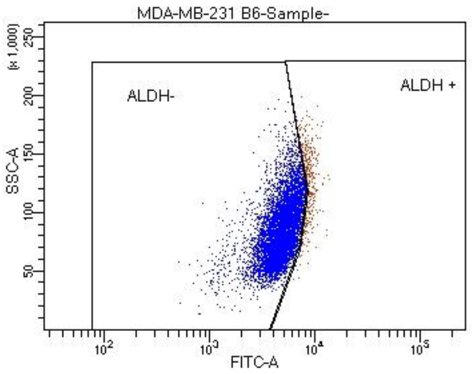

(o) **(p)**

Figure S6. Representative dot plots for ALDH1 results. Cells were treated with β1-conglutin (2.5 ng/µl), β3-conglutin (5 ng/µl) or β6-conglutin (10 ng/µl) for 24h in MCF10-A (a-d), MCF-7(e-h), SK-BR-3 (i-l) and MDA-MB-

321(m-p). After 24 hours of β1, β3, and β6-conglutin treatment, cells were incubated with BODIPY-amino acetaldehyde (BAAA), a fluorescent non-toxic substrate for ALDH, which was converted into BODIPY-aminoacate (BAA) and retained inside the cells. The specific inhibitor of ALDH, diethylaminobenzaldehyde (DEAB), was used to control for background fluorescence, shown as "Inhibitor" for each cell line. Viable ALDH1+ cells were quantified by flow cytometry on a BD FACS Aria IIIu Flow Cytometer (Becton Dickinson, BD Bioscience).

MCF-10A

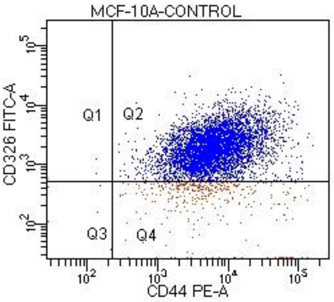

(a)

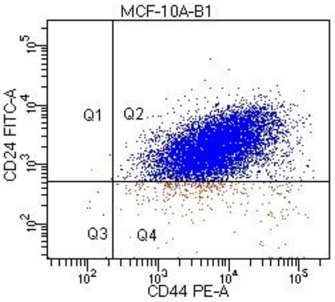

(b)

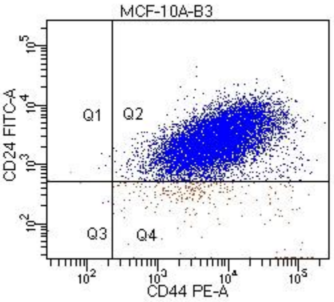

(c)

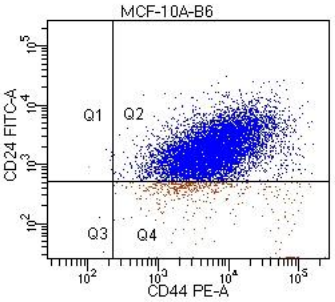

(d)

MCF-7

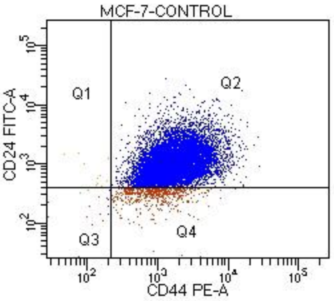

(e)

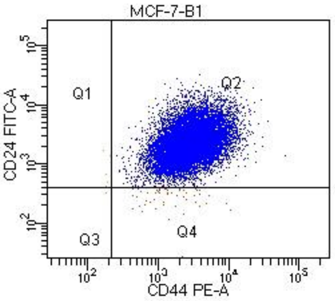

(f)

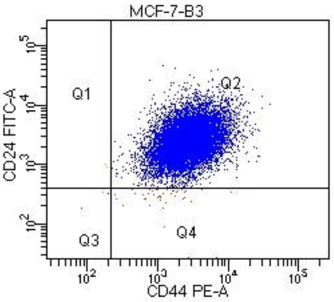

(g)

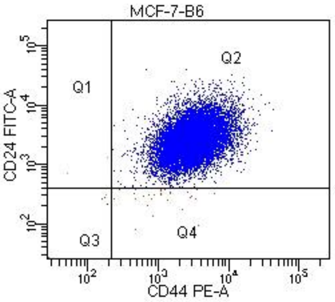

(h)

SK-BR-3

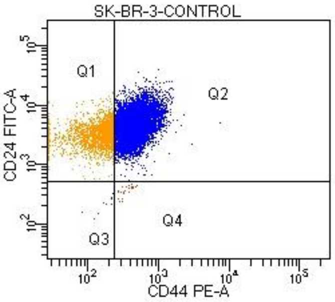

(i)

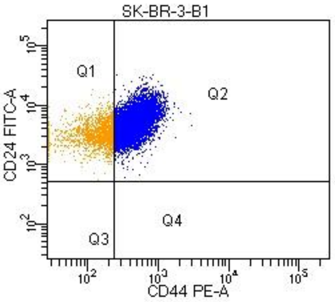

(j)

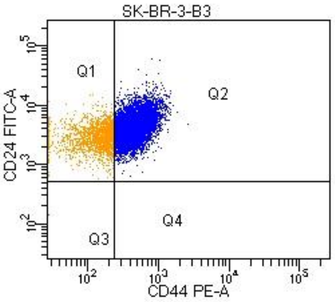

(k)

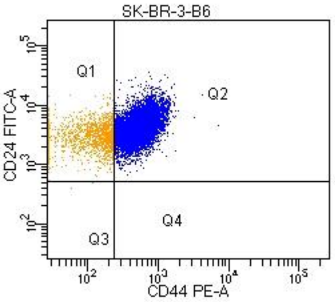

(l)

MDA-MB-231

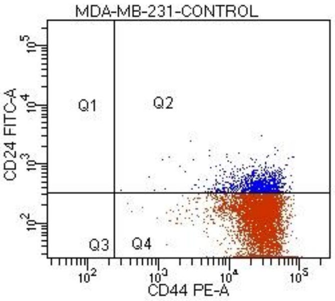

(m)

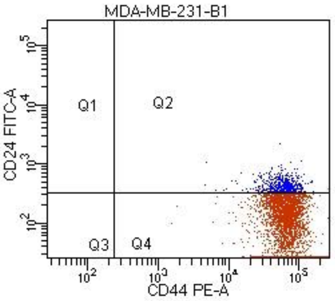

(n)

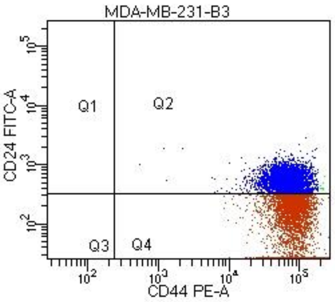

(o)

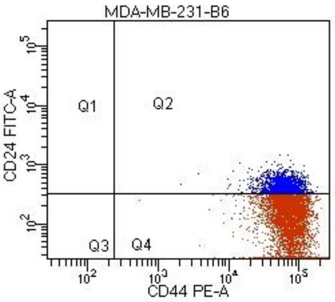

(p)

Figure S7. Representative dot plots for CD24/CD44 surface markers results. Cells were treated with β1-conglutin (2.5 ng/µl), β3-conglutin (5 ng/µl) or β6-conglutin (10 ng/µl) for 24h in MCF10-A (a-d), MCF-7(e-h), SK-BR-3 (i-l) and MDA-MB-321(m-p). After 24 hours of β1, β3, and β6-conglutin treatment, cells were incubated using CD44-PE and CD24-FITC antibodies (Biolegend, San Diego, CA, USA). After 30 minutes of incubation at darkness and at 4ºC, the samples were analyzed using a BD FACSAria IIIu flow cytometry (Becton Dickinson, BD Biosciences).

Table S1. Similarity matrix for paired β-conglutins treatments

	b1	b3	b6
b1	**1.000**	0.400	0.200
b3	0.400	**1.000**	0.800
b6	0.200	0.800	**1.000**

Data represent Pearson's correlation coeficients. Bold numbers indicate similarity. Similarity threshold were 0.95.

Table S2. Similarity matrix for paired cell lines treated

	MCF-10A	MCF-7	MDA-MB-231	SK-BR-3
MCF-10A	**1.000**	**1.000**	-1.000	-0.500
MCF-7	**1.000**	**1.000**	-1.000	-0.500
MDA-MB-231	-1.000	-1.000	**1.000**	0.500
SK-BR-3	-0.500	-0.500	0.500	**1.000**

Data represent Pearson's correlation coeficients. Bold numbers indicate similarity. Similarity threshold were 0.95.
